# Intra-articular steroid injections for lumbar disk herniation: a systematic review and meta-analysis

**DOI:** 10.1007/s00701-025-06676-4

**Published:** 2025-11-14

**Authors:** Saran Singh Gill, Pratik Ramkumar, Abith Ganesh Kamath, Sreeraag Kanakala, Akhil Anil, Srikar Reddy Namireddy, Srihan Yalavarthy, Daniele S. C. Ramsay, Ahmed Salih, Ahkash Thavarajasingam, Adrisa Prashar, Sajeenth Vishnu K, Tim Beutel, Salvatore Russo, Santhosh G. Thavarajasingam, Hariharan Subbiah Ponniah

**Affiliations:** 1 Imperial College London, London, UK; 2 Imperial Brain & Spine Initiative, Imperial College London, London, UK; 3 Faculty of Medicine, Medizinische Hochschule Hannover, Hannover, Germany; 4 Imperial College Healthcare NHS Trust, London, UK

**Keywords:** LDH, IA Injection, IESI, TFESI, CESI

## Abstract

**Introduction:**

Lumbar disc herniation (LDH) is one of the most common causes of lower back pain, radiculopathy, and functional impairment. Intra-articular (IA) steroid injections, including transforaminal (TFESI), interlaminar (IESI), and caudal (CESI) epidural steroid injections, are commonly administered to alleviate these symptoms when surgery is not indicated or opted for. This systematic review and meta-analysis evaluates the efficacy of these injection modalities in reducing pain and disability in LDH patients.

**Methods:**

Following PRISMA, 19,664 studies on IA steroid injections for LDH were screened, yielding 41 eligible studies. Random-effects and fixed effects meta-analyses computed pooled standardized mean changes (SMC), depending on heterogeneity (I^2^).

**Results:**

TFESI showed strong short-term efficacy, with the greatest pooled NRS improvement of -5.15 (95% CI: -6.59, -3.72, p < 0.001, I^2^ = 99.14%) at 3 months and the largest VAS reduction of -30.53 (95% CI: -43.89, -17.17, p < 0.001, I^2^ = 99.99%) at 3 months. CESI had the highest ODI improvement at 1 month (-18.99, 95% CI: -26.88, -11.10, p < 0.001, I^2^ = 99.35%), while IESI demonstrated the greatest ODI reduction at 6 months (-16.06, 95% CI: -16.83, -15.28, p < 0.001, I^2^ = 18.85%).

**Conclusion:**

This meta-analysis suggests that IA injections may relieve LDH symptoms, with TFESI showing the greatest pain relief and functional improvement. However, significant heterogeneity calls for standardized protocols and further research. Demographic factors minimally influenced outcomes, whereas methodological variability underscores treatment complexity. Future studies should emphasize methodological consistency and personalized approaches to optimize patient outcomes.

**Supplementary Information:**

The online version contains supplementary material available at 10.1007/s00701-025-06676-4.

## Introduction

Lumbar Disc Herniation (LDH), or lumbar herniated disc, is defined by the displacement of an intervertebral disc beyond its normal anatomical boundaries, frequently resulting in nerve compression and symptoms such as lower back pain, radiculopathy, weakness, or numbness in the lower extremities [38, 66]. LDH has an annual incidence of 5–20 cases per 1,000 individuals, with higher prevalence observed in males and those aged 30–50 years [25, 33]. Initial management typically includes exercise, physiotherapy, and analgesics, but refractory cases often require advanced interventions [4, 91]. For patients who decline surgery, seek to delay invasive procedures, or have self-limiting conditions like spontaneous regression of prolapsed discs, intra-articular (IA) epidural injections, in conjunction with physiotherapy, provide an effective option for symptomatic relief from LDH [3, 13, 37, 43, 64, 68].

Intra-articular (IA) steroid injections, including transforaminal epidural steroid injections (TFESI), interlaminar epidural steroid injections (IESI), and caudal epidural steroid injections (CESI), have emerged as promising treatment options, offering symptomatic relief for LDH patients [30, 56, 59, 69, 90]. IA steroid injections into the epidural space reduce inflammation by inhibiting the release of pro-inflammatory cytokines and chemokines, while inhibiting the production of cyclooxygenase-2 (COX-2) and prostaglandins (PGE2), while also decreasing matrix metalloproteinase (MMP) synthesis, thus alleviating pain, swelling, radiculopathy, and improving nerve function [51, 89]. However, despite their effectiveness, the optimal approach to epidural steroid injections to yield the best clinical outcomes for LDH patients remains a subject of ongoing debate.

This systematic review and meta-analysis critically evaluates the available evidence to provide a robust qualitative and quantitative synthesis of the relative efficacy of TFESI, IESI, and CESI in clinical practice. By synthesizing the existing literature, this study aims to support evidence-based recommendations, and improve the pain management and functional outcomes for patients with LDH. While prior research has explored the efficacy of steroid injections for LDH, this work represents the most comprehensive comparative analysis of these injection techniques to date [8, 39, 40, 57, 73]. Our objective was to assess and integrate the evidence for these three approaches, providing a quantitative foundation for informed clinical decision-making.

## Methodology

### Search strategy and study selection

**Fig. 1:**
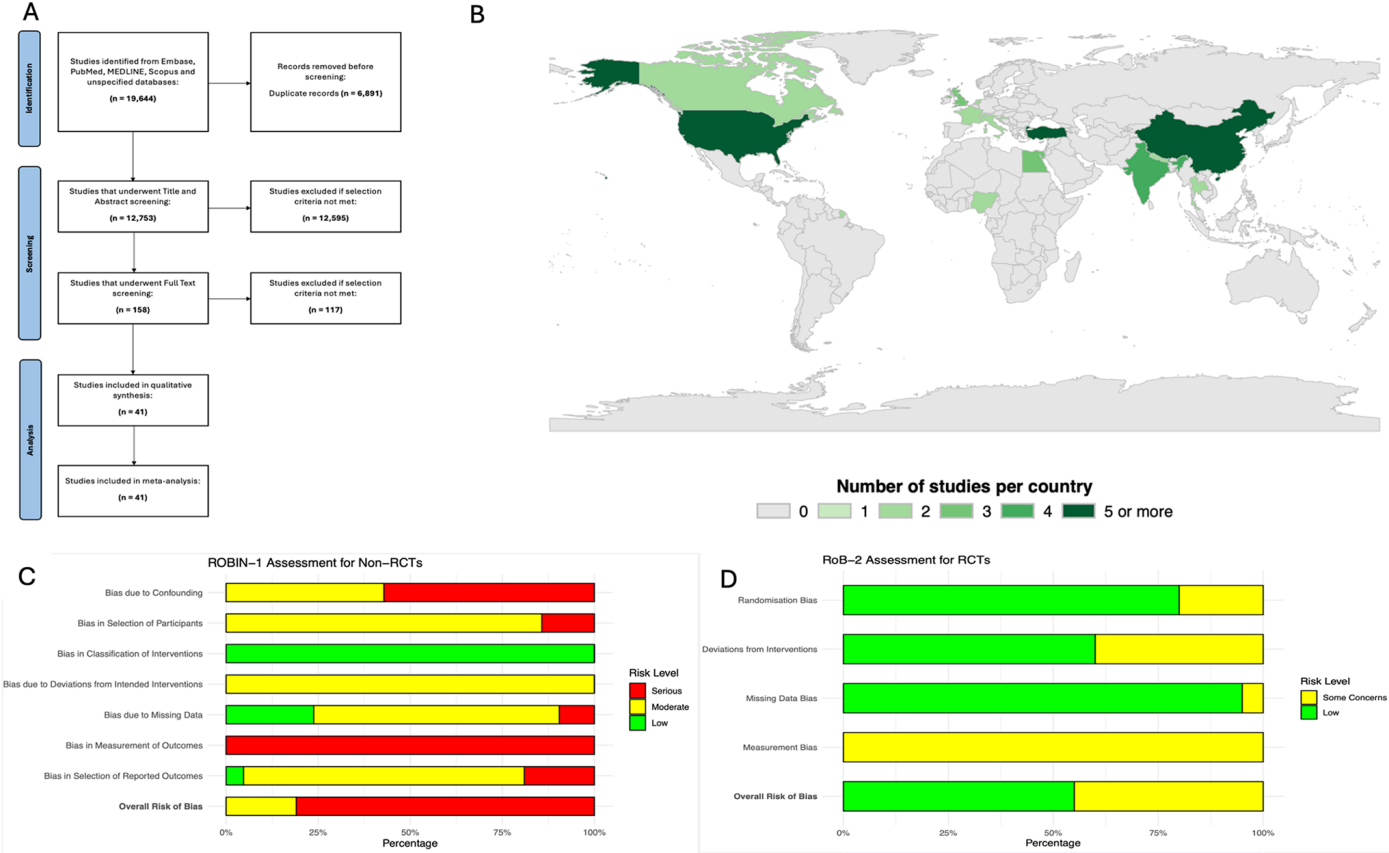
Study descriptors. **A**: PRISMA. **B**: World Map. **C**: ROBIN-1 Assessment for Non-RCTs. **D**: RoB-2 Assessment for RCTs

This study was registered on PROSPERO (CRD42025649369) and was conducted in accordance with PRISMA 2020 guidelines [60]. The literature search, conducted on May 17th 2024, across PubMed, MEDLINE, EMBASE, OVID, and Scopus, aimed to identify studies on IA injections for managing LDH. The detailed search strategy is available in Supplementary Table S1, with the PRISMA flowchart outlining the study selection process in Fig. 1. The inclusion criteria focused on original, peer-reviewed, quantitative studies published in English that examined the longitudinal efficacy of IA steroid injections for lumbar disc herniation (LDH) in adults, assessing outcomes such as pain reduction (Visual Analogue Scale and NRS) or functional improvement (ODI). Studies involving adolescents (< 18 years), reviews, abstracts, editorials, and case reports were excluded, as detailed in Supplementary Table S2. Title and abstract screening was performed using Covidence software for duplicate removal and independent review by four authors (AGK, SRN, SR, SY), followed by full-text assessment by three reviewers (AGK, AA, PR), with any disagreements resolved by consensus with SSG.

### Data extraction

Relevant data from each included study was manually extracted on independent spreadsheets, capturing information on study characteristics (title, authors, country of publication, publication date), type of IA injection, contents of the injection demographic details, performance metrics, and study conclusions. This was done by three independent researchers (AA, PR, AGK) For studies with missing data, corresponding authors were contacted for clarification. A detailed list of extracted variables is provided in Supplemental Digital Content 1: Supplementary Table S3, while key findings are summarized in Tables 1–3.

### Critical appraisal

Three independent reviewers (AA, PR, AGK) assessed the risk of bias for each study using two complementary tools tailored to study design: the Risk of Bias in Non-Randomised Studies of Interventions (ROBINS-I) for non-randomized studies and the Risk of Bias-2 (RoB-2) tool for randomized trials [77, 78]. ROBINS-I evaluates potential bias across seven domains, including confounding, participant selection, intervention classification, deviations from intended interventions, missing data, outcome measurement, and selective reporting, providing a comprehensive assessment of methodological quality in observational studies [77]. In contrast, RoB-2 focuses on five domains specific to randomized trials: randomization processes, deviations from intended interventions, missing outcome data, outcome measurement, and selective reporting, ensuring a rigorous evaluation of trial validity [78]. Together, these tools offer a systematic and robust framework for assessing the reliability and quality of diverse study designs. Methodological rigor and evidence quality were assessed using the Oxford Centre for Evidence-Based Medicine (OCEBM) Levels of Evidence framework and the GRADE framework, emphasizing the balance of benefits and harms to guide clinical decision-making (Supplemental Digital Content 1: Supplementary Tables 3–6) [10, 19].

### Statistical analysis

Statistical analyses were performed in R (Version 4.4.3) using the meta package. Pooled standardized mean changes (SMC) for VAS, ODI, and NRS were calculated across TFESI, IESI, and CESI at predefined timepoints. Random-effects models (DerSimonian–Laird method) were used by default, with fixed-effect models applied when heterogeneity was low (I^2^ < 50%). The standard deviation of change scores was calculated assuming a pre–post correlation coefficient (r = 0.5), as recommended in the Cochrane Handbook (Sect. 6.5.2.8), to avoid bias in variance estimation. Forest plots illustrated effect sizes with 95% confidence intervals (CIs), and heterogeneity was quantified using I^2^ and τ^2^ statistics. Publication bias was assessed with funnel plots and Egger’s test when an adequate number studies were available in each domain. Only timepoints with data from ≥ 3 studies were reported. Sensitivity analyses were performed by restricting to low–risk of bias studies, based on ROBINS-I or RoB-2 scores, and to RCTs only.

To explore factors influencing effect sizes and heterogeneity, a single-covariate meta-regression was performed for each outcome–intervention combination. Covariates included follow-up duration (months), sample size, mean age, mean BMI, and proportion of male participants. Models used restricted maximum likelihood (REML) estimation when heterogeneity was substantial (I^2^ ≥ 50%) and fixed-effect modelling when I^2^ was low. Regression coefficients, standard errors, 95% CIs, and p-values were reported. Analyses assumed (i) a linear relationship between covariates and effect sizes, (ii) independence of effect sizes across studies, (iii) accurate estimation of within-study variances (with change-score SDs calculated using r = 0.5), (iv) model choice validity based on I^2^, (v) no adjustment for additional covariates given the single-covariate design, and (vi) sufficient between-study variability in covariates to allow estimation.

## Results

A total of 19,644 studies were screened. Ultimately, 41 studies met the criteria and were included in this systematic review and meta-analysis (Fig. 1A). [1, 2, 6, 7, 11, 14, 16–18, 21–24, 27–29, 32, 34–36, 41, 45–48, 54, 58, 59, 61, 62, 70, 72, 74, 75, 80, 81, 83, 85–88] All of which were included in the meta analysis. These studies encompassed a combined sample of 6765 patients.

Study characteristics, including study design, country of origin, sample size, follow-up duration, and intervention type, are detailed in Table 1. Of the included studies, 25 (59.5%) investigated TFESI, 13 (31%) focused on CESI, and the rest focused on comparison between techniques, with some including IESI.

**Table 1:** Study Characteristics

Title	Author (Year)	Country	Study Design	Approach	Injection Composition
Comparison of treatment outcomes in lumbar disc herniation patients treated with epidural steroid injections: interlaminar versus transforaminal approach	Bensler et al. [7]	Swizterland	Retrospective Cohort	TFESI	40 mg triamcinolone acetonide (1ml), 0.2% ropivacaine (1ml)
				ILESI	40 mg triamcinolone acetonide (1ml), 0.2% ropivacaine (1ml)
CT-guided Pulsed Radiofrequency Combined with Steroid Injection for Sciatica from Herniated Disk: A Randomized Trial	Napoli et al. [54]	Italy/US	RCT	TFESI	20mg/ml lidocaine (1ml), 10mg/ml dexamethasone (2ml) or 40mg/ml triamcinolone acetonide (2ml)
				TFESI with Pulsed Radiofrequency	20mg/ml lidocaine (1ml), 10mg/ml dexamethasone (2ml) or 40mg/ml triamcinolone acetonide (2ml)
Ultrasound-Guided Transforaminal Injections of Platelet-Rich Plasma Compared with Steroid in Lumbar Disc Herniation: A Prospective, Randomized, Controlled Study	Xu et al. [87]	China	RCT	TFESI	Betamethasone (2ml), 0.9% sterile saline (0.5ml), 2% lidocaine (0.5ml)
"Platelet-Rich Plasma" epidural injection an emerging strategy in lumbar disc herniation: a Randomized Controlled Trial	Wongjarupong et al. [86]	Thailand	RCT	TFESI	1% lidocaine (2ml), 40 mg triamcinolone
Transforaminal Epidural Steroid Injection in the Treatment of Pain in Foraminal and Paramedian Lumbar Disc Herniations	Guclu et al. [28]	Turkey	Retrospective Cohort	TFESI for Foraminal LDH	4 mg dexamethasone, 0.33% lidocaine (3ml)
				TFESI for Paramedian LDH	4 mg dexamethasone, 0.33% lidocaine (3ml)
Evaluation of the effectiveness of transforaminal epidural steroid injection in far lateral lumbar disc herniations	Evran et al. [22]	Turkey	Retrospective Cohort	TFESI	40 mg methylprednisolone acetate (1ml), 10 mg bupivacaine hydrochloride (2ml)
Comparative effectiveness of lumbar transforaminal epidural steroid injections with particulate versus nonparticulate corticosteroids for lumbar radicular pain due to intervertebral disc herniation: a prospective, randomized, double-blind trial	Kennedy et al. [34]	US	RCT	TFESI	10mg/ml dexamethasone phosphate (1.5ml)
				TFESI	40 mg/ml triamcinolone acetonide (1.5 ml)
Effect of fluoroscopically guided caudal epidural steroid or local anesthetic injections in the treatment of lumbar disc herniation and radiculitis: a randomized, controlled, double blind trial with a two-year follow-up	Manchikanti et al. [46]	US	RCT	CESI	0.5% lidocaine (9ml), stereoid (1ml)
Transforaminal Epidural Steroid Injection Improves Neuropathic Pain in Lumbar Radiculopathy: A Prospective, Clinical Study	Sencan et al. [70]	Turkey	Prospective Cohort	TFESI	80 mg methylprednisolone acetate, physiological saline (1cc), 0.5% bupivacaine (1cc)
309 patients treated with fluoroscopy-guided caudal epidural injection for lumbar disc herniation	Akşan et al. [2]	Turkey	Prospective Cohort	CESI	0.5% of 40 mg bupivacaine hydrochloride (8ml), 4mg/ml dexamethasone (2ml), saline (10ml)
Optimal Timing and Outcome of Transforaminal Epidural Steroid Injection for the Management of Radicular Pain due to Extruded Lumbar Disc Herniation	Guclu et al. [27]	Turkey	Prospective Cohort	TFESI	0.33% lidocaine (3ml), 4 mg dexamethasone
Caudal epidural steroid injection versus transforaminal ESI for unilateral S1 radiculopathy: a prospective, randomized trial	Ozturk et al. [59]	Turkey	RCT	CESI	12 mg dexamethasone (3ml), 0.5% bupivacaine (1ml), saline (2ml)
				TFESI	12 mg dexamethasone (3ml), 0.5% bupivacaine (1ml), saline (1ml)
Comparison of the Effect of Single Lumbar Transforaminal Epidural Steroid Injections for the Treatment of L4-5 and L5-S1 Paramedian Disc Herniation	Adilay et al. [1]	Turkey	Retrospective Cohort	TFESI—L4-5	0.33% lidocaine (3ml),4mg dexamethasone
				TFESI—L5-S1	0.33% lidocaine (3ml),4mg dexamethasone
The role of fluoroscopic interlaminar epidural injections in managing chronic pain of lumbar disc herniation or radiculitis: a randomized, double-blind trial	Manchikanti et al. [47]	US	RCT	ILESI	0.5% lidocaine (5 ml), non-particulate betamethasone (1ml)
Fluoroscopically guided caudal epidural steroid injections for axial low back pain associated with central disc protrusions: a prospective outcome study	Lee et al. [41]	US	Prospective Cohort	CESI	80 mg triamcinolone, 1% lidocaine (6cc)
Lumbar retrodiscal versus post-ganglionic transforaminal epidural steroid injection for the treatment of lumbar intervertebral disc herniations	Park et al. [62]	South Korea	RCT	TFESI—retrodiscal	40 mg triamcinolone acetonide (1-2ml), 1% lidocaine (1-3ml)
				TFESI—post-ganglionic	40 mg triamcinolone acetonide (1-2ml), 1% lidocaine (1-3ml)
Epidural corticosteroid injections for sciatica due to herniated nucleus pulposus	Carette et al. [14]	Canada	RCT	TFESI	80 mg methylprednisolone acetate (2ml), isotonic saline (8ml)
Short-term assessment of periradicular corticosteroid injections in lumbar radiculopathy associated with disc pathology	Viton et al. [81]	France	Prospective Cohort	TFESI	2.4mg cortivazol
Effectiveness of epidural steroid injection for the management of symptomatic herniated lumbar disc	Baral et al. [6]	Nepal	Prospective Cohort	IESI	80 mg methylprednisolone, 0.5% bupivacaine (2ml), normal saline (8ml)
Transforaminal epidural injections in chronic lumbar disc herniation: a randomized, double-blind, active-control trial	Manchikanti et al. [45]	USA	RCT	TFESI	3 mg betamethasone (0.5ml), 1% lidocaine
The Outcome of Epidural Injections in Lumbar Radiculopathy Is Not Dependent on the Presence of Disc Herniation on Magnetic Resonance Imaging: Assessment of Short-Term and Long-Term Efficacy	Verheijen et al. [80]	Netherlands	Retrospective Cohort	TFESI	20-80mg methylprednisolone, 7.5-20mg dexamethasone lidocaine, chirocaine, or bupivacaine
Caudal epidural steroid injection for chronic low back pain: A prospective analysis of 107 patients	Dernek et al. [16]	Turkey	Retrospective Cohort	CESI	betamethasone sodium (2ml), saline (8ml)
Comparison of the effectiveness of lumbar transforaminal epidural injection with particulate and nonparticulate corticosteroids in lumbar radiating pain	Park et al. [61]	Korea	RCT	TFESI	7.5mg dexamethasone
				TFESI	40 mg triamcinolone acetate
Epidural steroid injection in patients with lumbosacral radiculopathy in Abuja, Nigeria	Kawu [35]	Nigeria	Prospective Cohort	TFESI	80 mg methylprednisolone acetate, 0.5% marcaine (4ml)
				ILESI	80 mg methylprednisolone acetate, 0.5% marcaine 0.5% (4ml)
Comparison of Epidural Steroid Injection Efficiency with Two Different Doses in Radiculopathies Associated with Lumbar Disc Herniation	Ozsoy-Unubol et al. [58]	Turkey	Prospective Cohort	TFESI	40 mg methylprednisolone
				TFESI	80 mg methylprednisolone
The Synergistic Effect of Combined Transforaminal and Caudal Epidural Steroid Injection in Recurrent Lumbar Disc Herniations	Evran et al. [24]	Turkey	Prospective Cohort	TFESI	40 mg methylprednisolone acetate (1 ml), 10 mg bupivacaine hydrochloride (2ml)
Functional Outcomes and Successful Predictors of Lumbar Transforaminal Epidural Steroid Injections (LTFESIs) for Lumbar Radiculopathy Under Fluoroscopic Guidance: A Prospective Study	Dhandapani et al. [17]	UK	Prospective Cohort	TFESI	4mg/ml dexamethasone (2ml), 0.25% bupivacaine (2ml)
Transforaminal Epidural Injection for Far Lateral Lumbar Disc Herniations: An Alternative to Surgery or Just a Delay?	Serifoglu and Etli [72]	Turkey	Retrospective Cohort	TFESI	40 mg methylprednisolone acetate (1 ml), 10 mg bupivacaine hydrochloride (2ml)
Microdiscectomy compared with transforaminal epidural steroid injection for persistent radicular pain caused by prolapsed intervertebral disc: the NERVES RCT	Wilby et al. [85]	UK	RCT	TFESI	20–60mg triamcinolone acetonide
Comparison of the Particulate Steroids, Betamethasone and Methylprednisolone, in Caudal Steroid Injection Under Ultrasound Guidance	Guler et al. [29]	Turkey	Retrospective Cohort	CESI	20mg/ml methylprednisolone (1ml), 0.5% bupivacaine (5ml), 0.9% NaCl (5ml)
				CESI	6mg/ml betamethasone (1ml), 0.5% bupivacaine (5ml), 0.9% NaCl (5ml)
Effectiveness of Epidural Steroid Injection Depending on Discoradicular Contact: A Prospective Randomized Trial	Budrovac et al. [11]	Croatia	RCT	TFESI	40 mg methylprednisolone, 0.25% levobupivacaine (5ml)
Transforaminal epidural steroid injection combined with radio frequency for the treatment of lumbar disc herniation: a 2-year follow-up	Wei et al. [83]	China	RCT	TFESI	80 mg methylprednisolone, 2% lidocaine (5ml), 1% ropivacaine (5ml)
Comparison Between a Single Subpedicular Transforaminal Epidural Steroid Injection and Lateral Recess Steroid Injection in Reducing Paracentral Disc Herniation–Related Chronic Neuropathic Leg Pain: A Retrospective Study	Jain et al. [32]	India	Retrospective Cohort	TFESI	0.25% bupivacaine (2ml), 750IU hyaluronidase, hypertonic saline (2ml), 8 mg dexamethasone
A randomized, double-blind, active-control trial of the effectiveness of lumbar interlaminar epidural injections in disc herniation	Manchikanti et al. [45]	US	RCT	IESI	0.5% preservative-free lidocaine (6 ml), non-particulate betamethasone (1ml)
Caudal epidural steroid injection ultrasound-guided versus fluoroscopy-guided in treatment of refractory lumbar disc prolapse with radiculopathy	Elashmawy et al. [21]	Egypt	RCT	CESI	40mg/ml triamcinolone acetonide, 0.5% lidocaine
				CESI	40mg/ml triamcinolone acetonide, 0.5% lidocaine
THE EFFICACY OF TRANSFORAMINAL EPIDURAL STEROID INJECTION (TFESI) IN SINGLE LEVEL LUMBAR DISC HERNIATION	Evran [22]	Turkey	Retrospective Cohort	TFESI	40 mg methylprednisolone acetate (1ml), 10mg bupivacaine hydrochloride (2ml)
Long-Term comparative study between transforaminal and interlaminar epidural injection of steroids in lumbar radiculopathy due to single-level disc herniation	Soliman and Fahmy [75]	Egypt	RCT	TFESI	betamethasone (2ml), bupivacaine (2ml)
				IESI	betamethasone (2ml), lidocaine (2ml)
Efficacy of Caudal Epidural Steroid Injection with Targeted Indwelling Catheter and Manipulation in Managing Patients with Lumbar Disk Herniation and Radiculopathy: A Prospective, Randomized, Single-Blind Controlled Trial	Yin et al. [88]	China	RCT	CESI with Indwelling Catheter	10 mg triamcinolone acetate, normal saline (40ml), lidocaine (5ml)
				CESI	10 mg triamcinolone acetate, normal saline (40ml), lidocaine (5ml)
Transforaminal epidural steroid injection combined with pulsed radio frequency on spinal nerve root for the treatment of lumbar disc herniation	Ding et al. [18]	China	Retrospective Cohort	TFESI	5 mg compound betamethasone, 2% lidocaine (5ml), 1 mg VitB12, normal saline (2ml)
Selective nerve root blocks vs. caudal epidural injection for single level prolapsed lumbar intervertebral disc – A prospective randomized study	Singh et al. [74]	India	RCT	CESI	80 mg methylprednisolone (2ml), 2% lignocaine (10ml), normal saline (20ml)
Outcome of single level disc prolapse treated with transforaminal steroid versus epidural steroid versus caudal steroids	Kamble et al. [34]	India	RCT	TFESI	40 mg triamcinolone acetate, bupivacaine (1ml), lignocaine (2ml)
				IESI	40 mg triamcinolone acetate, bupivacaine (1ml), lignocaine (1ml), normal saline (10ml)
				CESI	40 mg triamcinolone acetate, bupivacaine (1ml), lignocaine (2ml), normal saline (10ml)

Table 1 describes the approach (TFESI/ILESI/CESI) utilised in each study, along with the injection composition used to treat all included patients

**Table 2:** Results for all included studies highlighting changes in ODI, VAS, NRS and SLR following ESI therapy

Author (Year)	Approach	Sample Size (n)	Male (%)	Mean Age (SD)	Mean BMI (SD)	ODI (SD)	VAS (SD)	NRS (SD)	SLR (SD)
Bensler et al. [7]	TFESI	99	56.6	33.68 (8.209)				Baseline—5.84 (2.02) 1 month—4.16 (0.58)	
	ILESI	99	47.5	33.68 (5.441)				Baseline—6.23 (2.1) 1 month—4.13 (0.58)	
Napoli et al. [54]	TFESI	177	64	54 (16)	25.7 (3.4)	Baseline—54.7 (15.1) 1 month—36.2 (1.7) 3 months—29 (1.8) 12 months—24.8 (1.9)		LegNRS: Baseline—7.9 (1.1) 1 month—4.4 (0.2) 3 months—4.1 (0.2) 12 months—3.9 (0.2)	
	TFESI with Pulsed Radiofrequency	174	63	55 (16)	24.7 (3.5)	Baseline—52 (18.4) 1 month—20.3 (1.5) 3 months—14.5 (1.58) 12 months—12.3 (1.6)		LegNRS: Baseline—8.1 (1.1) 1 month—2.3 (0.2) 3 months—1.7 (0.2) 12 months—1 (0.2)	
Xu et al. [87]	TFESI	63	58.7	*56.0 (50.0–59.0)		*Baseline—27.0% (21.0–43.0) 1 month—18.0% (12.0–29.0) 3 month—20.0% (12.0–29.0) 6 month—20.0% (16.3–29.0) 12 months—20.0% (17.3–40.0)	*Baseline—60.0 (50.0–70.0) 1 month—30.0 (30.0–50.0) 3 month—30.0 (20.0–40.0) 6 month—20.0 (20.0–30.0) 12 months—20.0 (10.0–30.0)		
Wongjarupong et al. [86]	TFESI	15	53.3	39.13 (7.21)	25.55 (4.15)	Baseline—43.13 (10.81) 6 weeks—27 (13.3) 3 months—21.64 (7.92) 6 months—21.3 (5.21)	LegVAS: Baseline—59.67 (19.68) 6 weeks—36.36 (16.9) 3 months—32 (11.35) 6 months—30 (7.45) BackVAS: Baseline—73 (13.86) 6 weeks—35 (17.18) 3 months—29 (13.08) 6 months—28.5 (10.29)		
Guclu et al. [28]	TFESI for Foraminal LDH	370	43.8	53.3 (11.35)			Baseline—67.11 (4.28) 3 months—34.78 (3.64)		
	TFESI for Paramedian LDH	1262	36.7	56.4 (9.87)			Baseline—62.16 (6.65) 3 months—19.07 (4.5)		
Evran et al. [24]	TFESI	32	46.25	45 (11.63)		Baseline—52.38 (6.84) 3 months—37.41 (14.1) 6 months—34.88 (14.33)	Baseline—86.3 (5.5) 3 months—50.9 (8.5) 6 months—45.6 (16.6)		
Kennedy et al. [36]	TFESI	41	65.9	35.9 (-)				Baseline—7.95 (-) 3 months—1.61 (-) 6 months—1.37 (-)	
	TFESI	37	64.9	35.6 (-)				Baseline—6.86 (-) 3 months—1.77 (-) 6 months—1.25 (-)	
Manchikanti et al. [46]	CESI	60	38	43 (14.5)		Baseline—27.9 (4.8) 3 months—13.6 (6.5) 6 months—13.7 (7) 12 months—13.1 (7) 18 months—13.2 (6.7) 24 months—13.5 (7.2)		Baseline—7.8 (0.9) 3 months—3.4 (1.7) 6 months—3.5 (1.7) 12 months—3.5 (1.9) 18 months—3.5 (1.8) 24 months—3.6 (1.8)	
Sencan et al. [70]	TFESI	58	56.9	41.6 (11.1)	33.4 (-)	*Baseline—48 (34–60) 3 months—22 (12–41)		*Baseline—8 (7–9) 3 months—3 (0–5)	
Akşan [2]	CESI	309	32.4	56.5 (-)			Baseline—83.9 (7.15) 1 month—24.15 (13.6) 12 month—22.65 (13.05)		
Guclu et al. [27]	TFESI	305	65.9	43.3 (7.83)			Baseline—87.65 (5.59) 3 months—22.81 (4.01)		
Ozturk et al. [59]	CESI	30	60	41.86 (-)	27.14 (1.55)	Baseline—43.86 (8.45) 3 months—26.46 (8.29)		Baseline—7.43 (0.73) 1 month—2.5 (1.1) 3 months—2.93 (1.78)	
	TFESI	30	50	38.86 (-)	26.81 (2.64)	Baseline—45.26 (7.92) 3 months—26.4 (9.43)		Baseline—7.56 (0.77) 1 month—2.6 (1.21) 3 months—3.1 (1.76)	
Adilay et al. [1]	TFESI—L4-5	593	40.8	56.5 (9.9)			LegVAS: Baseline—63.09 (5.37) 3 months—15.81 (3.58)		
	TFESI—L5-S1	504	41.7	58.4 (9.98)			LegVAS: Baseline—61.15 (5.45) 3 months—27.06 (3.62)		
Manchikanti et al. [47]	ILESI	60	62	40.6 (12.5)		Baseline—29.6 (5.2) 3 months—14 (4.2) 6 months—13.5 (4.2) 12 months—13 (4.2)		Baseline—8 (1) 3 months—3.5 (1) 6 months—3.5 (1) 12 months—3.4 (1.2)	
Lee et al. [41]	CESI	68	61.8	41 (12)				Baseline—4.53 (0.27) 1 month—2.69 (0.34) 3 months—1.18 (0.38) 6 months—3.22 (0.41) 12 months—2.05 (0.48)	
Park et al. [62]	TFESI—retrodiscal	20	60	42.8 (13.2)			Baseline—75 (13) 1 month—31 (16) 2 months—35 (15)		
	TFESI—post-ganglionic	20	45	48.9 (13.5)			Baseline—67 (19) 1 month—31 (16) 2 months—30 (16)		
Carette et al. [14]	TFESI	78	71.8	39 (9.3)		Baseline—49.6 (16.7) 3 months—32.3 (20.6)			
Viton et al. [81]	TFESI	40	47.5				Baseline—49.7 (-) 10 days—30.7 (-) 3 months—23.8 (-)		
Baral et al. [6]	IESI	62	52	41.04 (13.26)		Baseline—60.86 (-) 1 month—40.48 (-) 6 month—35.68 (-)	Baseline—69.8 (-) 1 month—43.6 (-) 6 month—36.8 (-)		
Manchikanti et al. [45]	TFESI	55	45	42.6 (11.2)	26.8 (5.7)	Baseline—29.9 (4.8) 3 months—14.7 (6.4) 6 months—14.3 (6.6) 12 months—14.5 (6.6) 18 months—14.3 (6.5) 24 months—14.1 (6.5)		Baseline—8.2 (0.9) 3 months—4.1 (1.8) 6 months—3.9 (1.5) 12 months—3.9 (1.6) 18 months—4 (1.7) 24 months—4 (1.6)	
Verheijen et al. [80]	TFESI	337	48.1	55.12 (15.28)				Baseline—8 (1.4) 2 months—4.7 (2.8)	
Dernek et al. [16]	CESI	107	19.6	48 (8.2)		Baseline—32.7 (1) 3 months—23.7 (7.7) 6 months—23.7 (7.7)	Baseline—78 (6) 3 months—37 (23) 6 months—37 (23)		
Hong et al. (2010)	TFESI	53	49.1	55.5 (14.9)			Baseline—74 (14) 1 month—41 (19)		
	TFESI	53	45.3	62.5 (10.8)			Baseline—83 (9) 1 month—24 (9)		
Kawu [35]	TFESI	25	72	46.9 (10.7)		Baseline—62.4 (7.4) 6 months—20.8 (3)	Baseline—78.4 (10.6) 3 months—28.6 (5.6)		
	ILESI	24	66.7	48.3 (11.3)		Baseline—60.7 (6.7) 6 months—43.2 (4.4)	Baseline—76.8 (10.2) 3 months—50.6 (8.3)		
Ozsoy-Unubol et al. [58]	TFESI	33	33.3	48 (11.7)	27.6 (5.2)	Baseline—54.7 (11.9) 3 months—34.1 (7)		Baseline—7.8 (1.7) 3 months—3.7 (2.1)	
	TFESI	34	26.5	51.4 (15.7)	28 (4.2)	Baseline—54 (16.9) 3 months—35.9 (19.7)		Baseline—7.9 (1.5) 3 months—4.3 (2.6)	
Evran et al. [24]	TFESI	71	19.72	49.7 (12.46)		Baseline—55.81 (8.04) 3 months—37.5 (8.39) 6 months—49.04 (7.13)	LegVAS: Basline—78.4 (7.7) 3 months—54 (13.2) 6 months—61.9 (10.06) BackVAS: Baseline—80.6 (12.4) 3 months—48.8 (13.6) 6 months—59.7 (11.5)		
Dhandapani et al. [17]	TFESI	52	51.92	43.22 (9.97)		Baseline—56.61 (8.97) 1 month—30.69 (11.03) 3 months—26.83 (8.76) 6 months—25.5 (10.4)			
Serifoglu and Etli [72]	TFESI	42	45.23	51.9 (11.63)		Baseline—61.29 (6.72) 1 month—35.49 (3.96) 2 months—29.21 (11.2) 3 months—16.88 (11.25)	Baseline—85.8 (6.3) 1 month—41.3 (13.5) 2 months—38.9 (7.9) 3 months—28.9 (18.7)		
Wilby et al. [85]	TFESI	80	50	41.2 (8.6)	27.2 (6.4)	Baseline—53.74 (19.35) 3 months—30.02 (24.38)			
Guler et al. [29]	CESI	40	42.9	48.09 (10.91)	24.47 (1.76)	Baseline—46.71 (16.15) 1 month—30.8 (17.65)	Baseline—87.6 (7.6) 1 month—31.2 (13)		
	CESI	40	36.8	47.52 (9.43)	24.4 (1.93)	Baseline—49.84 (9.11) 1 month—22.84 (6.44)	Baseline—88.4 (7.6) 1 month—47.3 (23.2)		
Budrovac et al. [11]	TFESI	59	32	33 (-)		Baseline—61 (-) 1 month—54 (-) 3 months—53 (-)	Baseline—70 (-) 1 month—40 (-) 3 months—40 (-)		
Wei et al. [83]	TFESI	110	42.7	64.7 (14.23)	24.48 (5.05)	Baseline—69.45 (6.71) 1 month—14.9 (3.9) 3 months—16.12 (4.39) 6 months—26.12 (6.05) 12 months—26.11 (8.92) 24 months—27.77 (8.46)	Baseline—70.4 (10.2) 1 month—21 (9.6) 3 months—23.8 (9.7) 6 months—26.6 (10.7) 12 months—32.7 (10.2) 24 months—33.3 (9.3)		
Jain et al. [32]	TFESI	126	44.4	45 (8.08)		Baseline—56 (-) 1 month—42 (-) 3 months—44 (-) 6 months—45 (-)		Baseline—7 (-) 1 month—4 (-) 3 months—4 (-) 6 months—5 (-)	
Manchikanti et al. [45]	IESI	60	62	40.6 (12.5)		Baseline—29.6 (5.2) 3 months—14 (4.2) 6 months—13.5 (4.2) 12 months—13 (4.2) 18 months—13.3 (5) 24 months—13.5 (4.8)		Baseline—8 (1) 3 months—3.5 (1) 6 months—3.5 (1) 12 months—3.4 (1.2) 18 months—3.6 (1.3) 24 months—3.7 (1.4)	Baseline—3.33 (1.11)
Elsjmawy et al. (2021)	CESI	59	42.4	42.53 (10.3)	30.63 (4.02)	Baseline—55.59 (7.39) 1 month—21.15 (16.21) 3 months—23.77 (20.22)	Baseline—70.31 (7.49) 1 month—23.85 (21.07) 3 months—27.73 (24.99)		Baseline—3.23 (1.08) 1 month—6.16 (1.55) 3 months—5.98 (1.81)
	CESI	62	41.9	42.69 (10.48)	30.45 (3.92)	Baseline—55.73 (6.54) 1 month—22.04 (16.78) 3 months—25.82 (21.07)	Baseline—71.69 (7.49) 1 month—24.03 (23.51) 3 months—26.84 (2.53)		Baseline—3.33 (1.11) 1 month—6.11 (1.64) 3 months—5.91 (1.87)
Evran [22]	TFESI	337	40.9	46.38 (11.6)		Baseline—51.3 (6.14) 3 months—30.69 (12.76) 6 months—48.65 (5.69)	Baseline—83.5 (7.5) 3 months—38.9 (18.5) 6 months—72 (9.4)		
Soliman and Fahmy [75]	TFESI	20	30	37.5 (10.822)		Baseline—35.34 (6.198) 3 months—17.78 (6.354)	Baseline −76.5 (8.14) 3 months—41.75 (13.49)		
	IESI	20	55	35 (9.937)		Baseline—32.99 (7.434) 3 months—19.49 (11.239)	Baseline—71.75 (8.375) 3 months—41.5 (18.995)		
Yin et al. [88]	CESI with Indwelling Catheter	43	65.1	55.18 (12.51)		Baseline—27.33 (7.88) 1 month—15.19 (11.25)	Baseline—54.05 (23.64) 1 month—29.15 (24.56)		
	CESI	42	33.3	55.19 (12.28)		Baseline—27.51 (6.3) 1 month—18.77 (8.9)	Baseline—58 (21) 1 month—37.05 (20.52)		
Ding et al. [18]	TFESI	45	71.1	60.32 (4.48)		Baseline—49.26 (12.18) 1 month—35.58 (7.97) 3 months—31.18 (24.52) 6 months—29.39 (4.67)			
Singh et al. [74]	CESI	40		36.98 (11.3)		Baseline—78.15 (5.4) 1 month—31.55 (8.7) 3 months—27.6 (11.8) 6 months—27.2 (13.9) 12 months—27 (14.1)	Baseline—74.2 (6) 1 month—28.5 (7) 3 months—30 (12) 6 months—29.3 (23) 12 months—31 (15)		
Kamble et al. [34]	TFESI	30				Baseline—37.7 (2.83) 1 month—16.3 (3.74) 6 months—16.8 (2.53)	Baseline—71 (7) 1 month—24 (9) 6 months—26 (7)		
	IESI	30				Baseline—36.9 (2.82) 1 month—22.1 (5.18) 6 months—21.4 (6.08)	Baseline—70 (7) 1 month—35 (12) 6 months—34 (14)		
	CESI	30				Baseline—38.3 (2.78) 1 month—22.4 (4.55) 6 months—22.4 (3.35)	Baseline—72 (6) 1 month—36 (11) 6 months—35 (10)		
*Median (Interquartile range)									

Table 2 reports the changes in in ODI, VAS, NRS and SLR from pre-ESI therapy to post-ESI therapy at different follow-ups

**Fig. 2:**
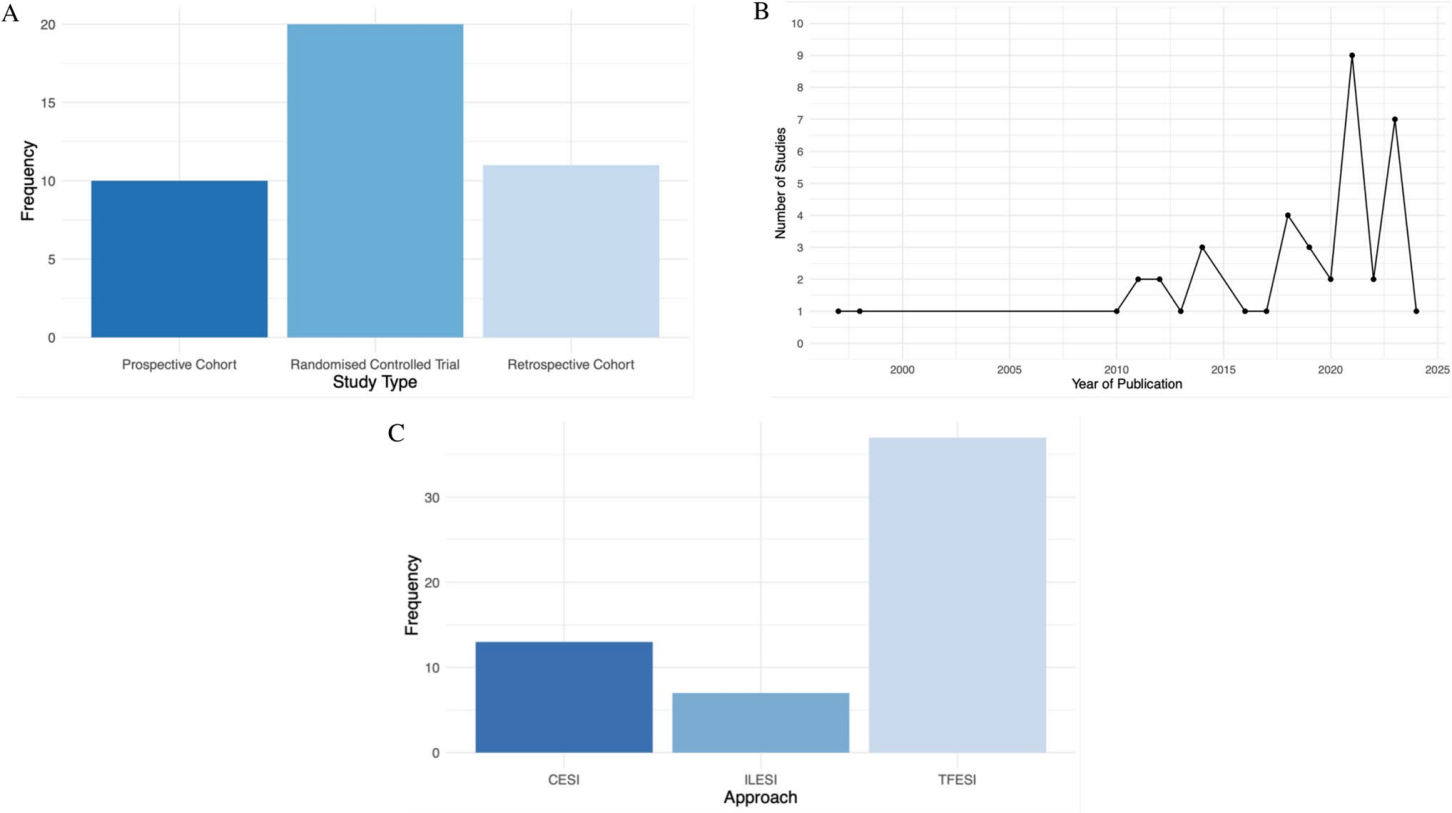
Summary Plots. **A**: Frequency of the Different Study Types Included. **B**: Yearly Trend of Publications from 1997 to 2024. **C**: Frequency of the Different Approaches Taken Across Included Studies

Using the ROBINS-I tool, 17/21 (81.0%) of the non-randomised studies were rated as "serious" overall (Fig. 2c). Among the RCTs assessed using RoB-2, 9/20 (45.0%) studies showed "some concerns" and 11/20 (55.0%) studies demonstrated "low" risk of bias (Fig. 2d). Evidence levels, as determined using the OCEBM criteria, classified the studies as level 2 (n = 20/41 (48.8%)), level 3 (n = 21/41 (51.2%)). GRADE assessments categorized 6/41 (14.6%) studies as having “ high”, 19/41 (46.3%) studies as "moderate" and with the 15/41 (36.6%) studies as "low" overall evidence quality. Detailed results of these evaluations are presented in Supplementary Tables 3–6.

### Transforaminal epidural steroid injections

25 studies analysed TFESI for LDH, exploring a variety of techniques and injection compositions. Across the studies, TFESI demonstrated a significant reduction in pain and disability, with improvements in functional and patient reported outcome measures (PROMs).

### Pain and disability reduction

All 25 studies reported substantial reductions in pain and disability, as measured by the VAS, ODI, NRS and SLR test. Xu et al. compared the effects of ultrasound-guided transforaminal injections of Platelet-Rich Plasma (PRP). The VAS for pain significantly improved in both groups, decreasing from 6.0 (baseline) to 2.0 (p < 0.001) at 1-year post-treatment, with no significant intergroup differences. The ODI also showed notable improvements, dropping from 35.0% (PRP) and 27.0% (steroid) at baseline to 19.0% and 20.0% at 1 year (p < 0.001), respectively. Similarly, in Dhandapani et al., the ODI significantly improved, with a reduction from 56.61% (pre-injection) to 25.5% at six months post-injection (p < 0.0001), indicating sustained functional benefits in 82.69% of patients. Overall, the included studies demonstrated significant and sustained improvements in pain and functional outcomes following injection therapy for lumbar disc herniation, as shown by reductions in VAS, ODI, and NRS scores.

### Variability in doses of corticosteroids

Some studies compared different doses of corticosteroids administered through TFESI. Namely, Ozsoy-Unobol et al. prospectively compared the use of 40mg and 80mg methylprednisolone whereby the ODI significantly improved in both groups (p < 0.001), but the 40 mg group had significantly lower ODI scores at the 3-week follow-up (p = 0.036). Hence, suggesting that while both 40 mg and 80 mg provided significant pain relief and functional improvement, the 40 mg dose demonstrated slightly better short-term outcomes and is favored for its lower risk of side effects.

### Particulate versus non-particulate corticosteroids

Two studies focused on specifically comparing the effects of particulate and non-particulate steroids in managing lumbar radicular pain due to LDH. Kennedy et al. compared the efficacy of particulate (triamcinolone) versus nonparticulate (dexamethasone). At 3 months, 73.2% (dexamethasone) and 73.0% (triamcinolone) of patients achieved ≥ 50% pain reduction, with similar results at 6 months (73.2% vs. 75.7%). However, the dexamethasone group required significantly more repeat injections, with 17.1% requiring three injections compared to only 2.7% in the triamcinolone group (p = 0.0052). In Park et al. triamcinolone demonstrated superior pain relief, with VAS scores improving from 8.3 ± 0.9 to 2.4 ± 0.9 at 1 month, compared to 7.4 ± 1.4 to 4.1 ± 1.9 in the dexamethasone group (p = 0.000). Therefore, our analysis found that both particulate and non-particulate steroids provided significant pain relief, but triamcinolone was more effective and required fewer repeat injections than dexamethasone.

### Caudal epidural steroid injections

Thirteen studies encompassing evaluating CESI for LDH with or without associated radiculopathy highlighted consistent benefits in pain reduction, disability improvement, and neural mobility.

### Pain and disability reduction

Similar to TFESI, all 13 studies reported substantial reductions in pain and disability, as measured by the VAS, ODI, SLR and NRS tests. Aksan et al. assessed the efficacy of fluoroscopy-guided caudal epidural injections in 309 patients with LDH. The VAS scores showed significant pain reduction, from 83.90 ± 7.15 at baseline to 24.15 ± 13.6 at 1 month (p = 0.04) and 22.65 ± 13.05 at 1 year (p = 0.04). The procedure was well tolerated, with only 3.56% of patients experiencing complications, all of which resolved without long-term sequelae. Dernek et al. also demonstrated significant reductions in VAS, from 7.8 ± 0.6 at baseline to 3.7 ± 2.3 at 3- and 6-months post-injection (p = 0 < 001). Similarly, Oswestry ODI scores improved, decreasing from 32.7 ± 1.0 at baseline to 23.7 ± 7.7 at both 3 and 6 months (p = 0.000). Overall, studies on caudal epidural injections for LDH consistently reported significant pain and disability reduction, with improvements sustained for up to a year and minimal complications.

### Fluoroscopy and catheter based techniques

A key theme in this group was the technique of administration. Yin et al. found that ESI with a targeted indwelling catheter provided superior short-term pain relief compared to traditional ESI. The Catheter Group had significantly lower VAS (back) scores at 1, 3, and 7 days post-injection (p < 0.05), but by day 28, the difference was no longer significant (p = 0.150). Similarly, ODI and JOA scores showed greater improvement at day 1 (p < 0.05), but no differences beyond this time point.

Elashmawy et al. compared ultrasound-guided (US) versus fluoroscopy-guided (FL) caudal ESI for lumbar disc prolapse with radiculopathy. Both techniques significantly reduced pain, with VAS scores dropping from ~ 70 at baseline to ~ 24 at 1 month (p < 0.001). ODI scores improved from ~ 55 to ~ 21 at 1 month (p < 0.001). However, no significant differences were observed between US and FL at any time point (p > 0.05).

The results show that injection techniques found that a targeted indwelling catheter provided superior short-term pain relief compared to traditional ESI, while ultrasound- and fluoroscopy-guided caudal ESI were equally effective in reducing pain and disability.

### Comparative studies

Several studies compared different steroid injection modalities, including TFESI, CESI and ILESI. Namely Soliman et al. compared the effectiveness of interlaminar versus transforaminal ESI. VAS scores for leg pain improved significantly in the transforaminal group at 48 weeks (p = 0.008), while no significant difference was found in back pain relief (p = 0.167). ODI scores improved more in the transforaminal group at both 12 weeks (p = 0.025) and 48 weeks (p = 0.03), indicating better functional recovery. Similarly, Ozturk et al. compared CESI and TFESI. Both groups showed significant pain relief, with NRS-11 decreasing from ~ 7.5 to ~ 2.9 in CESI and ~ 3.1 in TFESI at 3 months (p < 0.001). Treatment success rates were similar (CESI: 77%, TFESI: 73%, p = 0.766), indicating both techniques are equally effective. Fluoroscopy time (p = 0.024) and radiation exposure (p = 0.009) were significantly lower in CESI, favouring it as a safer alternative. Studies comparing different steroid injection modalities found that while all provided significant pain relief and functional improvement, transforaminal ESI offered superior long-term outcomes, whereas CESI and TFESI were equally effective, with CESI having lower radiation exposure and fluoroscopy time.

### Meta analysis

The overall pooled SMC for each scoring system reflected a positive overall effect of intra-articular treatments on pain reduction and functional improvement up to two years However, heterogeneity was substantial in the majority of outcomes, warranting closer examination of the individual modalities and their variability.

### Transforaminal epidural steroid injection (TFESI)

TFESI demonstrated substantial and sustained efficacy across pain and disability measures. For pain, measured by the NRS, significant improvements were observed at 1 month (−3.99, 95% CI: −5.64, −2.33, p < 0.001, I^2^ = 99.57%) and 3 months (−5.15, 95% CI: −6.59, −3.72, p < 0.001, I^2^ = 99.14%). Disability, assessed via the ODI, also improved markedly: at 1 month (−23.82, 95% CI: −35.49, −12.16, p < 0.001, I^2^ = 99.66%), 3 months (−23.33, 95% CI: −30.73, −15.94, p < 0.001, I^2^ = 99.42%), and 6 months (−21.13, 95% CI: −31.16, −11.11, p < 0.001, I^2^ = 99.77%). VAS scores showed significant reductions at 1 month (−4.10, 95% CI: −4.96, −3.24, p < 0.001, I^2^ = 83.66%), 6 weeks (−4.37, 95% CI: −5.43, −3.30, p < 0.001, I^2^ = 96.56%), 3 months (−30.53, 95% CI: −43.89, −17.17, p < 0.001, I^2^ = 99.99%), and 6 months (−15.19, 95% CI: −17.49, −12.88, p < 0.001, I^2^ = 99.76%). The 2-month, 1-year ODI, 2-year ODI, NRS 6-month, and NRS 18-month results were excluded as they were based on fewer than three studies. Across all included measures, heterogeneity was generally high, suggesting variation in effect sizes across studies, but the direction of effect consistently favoured TFESI. Sensitivity analyses restricted to low risk of bias studies and to RCTs only both confirmed the robustness of these findings, showing consistent patterns.

### Caudal epidural steroid injection (CESI)

Significant improvements were observed in both pain and disability scores. The NRS at 3 months showed a pooled SMC of −4.06 (95% CI: −4.93, −3.19, p < 0.001, I^2^ = 95.46%), indicating a notable reduction in pain. The ODI demonstrated improvements across multiple timepoints: at 1 month, the SMC was −18.99 (95% CI: −26.88, −11.10, p < 0.001, I^2^ = 99.35%), at 3 months −17.44 (95% CI: −23.68, −11.21, p < 0.001, I^2^ = 99.00%), and at 6 months −10.88 (95% CI: −17.27, −4.50, p < 0.001, I^2^ = 99.40%), all suggesting a sustained reduction in disability. Pain reduction, measured via the VAS, was also significant: −11.67 (95% CI: −17.52, −5.82, p < 0.001, I^2^ = 99.91%) at 1 month, −4.63 (95% CI: −5.06, −4.19, p < 0.001, I^2^ = 82.05%) at 3 months, −4.29 (95% CI: −5.12, −3.47, p < 0.001, I^2^ = 93.72%) at 6 months, and −23.47 (95% CI: −41.51, −5.43, p = 0.01, I^2^ = 99.97%) at 1 year. The 6-week, 1-year ODI, and all NRS results other than 3 months were excluded as they were based on fewer than three studies. Sensitivity analysis reinforced these findings, with analyses restricted to low risk of bias studies and to RCTs only both confirming the robustness of the main results, showing consistent significance patterns with only minor variations in effect sizes.

### Interlaminar epidural steroid injection (IESI)

Available data focused on ODI outcomes. For ODI, heterogeneity was low at all included timepoints, so fixed-effects estimates are reported. At 3 months, the pooled SMC was −15.52 (95% CI: −16.36, −14.68, p < 0.001, I^2^ < 0.01%), and at 6 months −16.06 (95% CI: −16.83, −15.28, p < 0.001, I^2^ = 18.85%), indicating consistent and sustained improvement in disability. The low heterogeneity across these outcomes suggests a high degree of consistency in IESI treatment effects. The 1-month, 1-year, and all NRS results were excluded as they were based on fewer than three studies. Both sensitivity analyses yielded similar results.

### Comparative effectiveness of therapy modalities

**Fig. 3:**
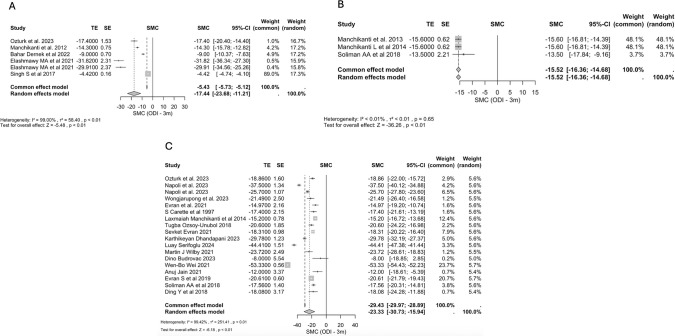
Forest Plots for ODI Comparing Baseline to 3 Months. **A**: CESI; **B**: IESI; **C**: TFESI

**Fig. 4:**
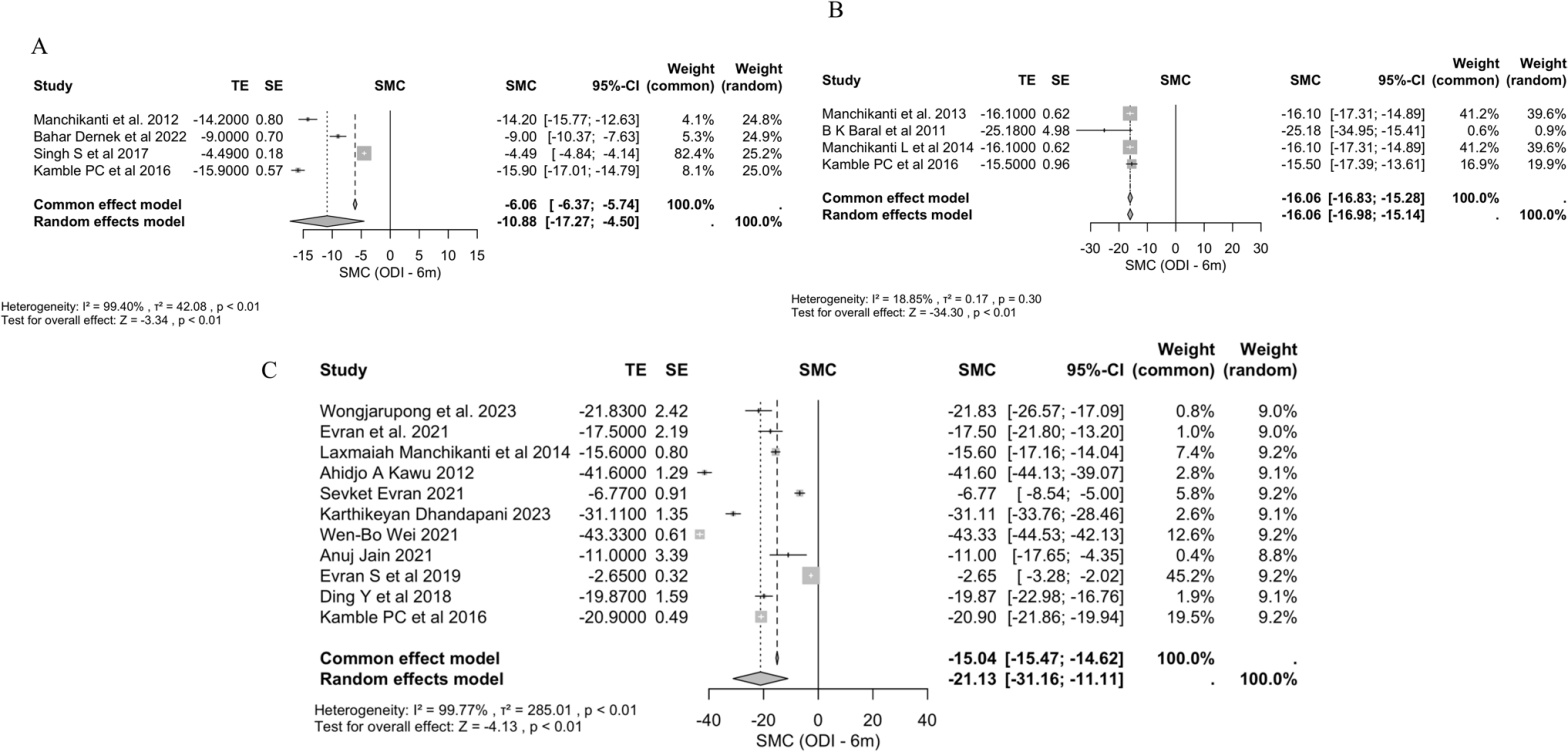
Forest Plots for ODI Comparing Baseline to 6 Months. **A**: CESI; **B**: IESI; **C**: TFESI

The pooled effect sizes differed substantially between the three treatment modalities. TFESI therapy demonstrated a larger pooled improvement in VAS, ODI, NRS and SLR scores compared to CESI and IESI (Figs. 3, 4). Significant heterogeneity was observed within all intervention groups. This underscores substantial variability in study protocols, patient populations, and outcome measures, making these findings less consistent and generalizable.

### Meta-regression results

For NRS, in the CESI group, higher mean age was associated with smaller improvements (β = –0.96, 95% CI –1.52 to –0.41, p < 0.01), whereas a greater proportion of male participants predicted larger effects (β = 5.80, 95% CI 0.42 to 11.17, p = 0.03). Larger sample sizes were also positively associated with effect size (β = 0.06, 95% CI 0.01 to 0.10, p = 0.01). In IESI, older age (β = –0.35, 95% CI –0.40 to –0.29, p < 0.01) and higher male proportion (β = –16.51, 95% CI –19.15 to –13.88, p < 0.01) were associated with smaller effects, whereas larger sample sizes were associated with greater effects (β = 0.06, 95% CI 0.05 to 0.07, p < 0.01). In TFESI, longer follow-up was linked to smaller improvements (β = –0.21, 95% CI –0.33 to –0.09, p < 0.01). For ODI, in TFESI, higher BMI was associated with larger improvements (β = 11.69, 95% CI 7.67 to 15.70, p < 0.01), while older age predicted smaller effects (β = –0.99, 95% CI –1.35 to –0.62, p < 0.01). In CESI, BMI showed a strong negative association with effect size (β = –3.23, 95% CI –4.37 to –2.09, p < 0.01). For VAS, in TFESI, higher BMI (β = –28.42, 95% CI –32.73 to –24.12, p < 0.01) and a greater proportion of male participants (β = –111.00, 95% CI –176.01 to –46.00, p < 0.01) were associated with smaller effects. In CESI, higher BMI was linked to larger improvements (β = 0.19, 95% CI 0.13 to 0.25, p < 0.01), while older age was also associated with smaller improvements (β = –1.46, 95% CI –2.63 to –0.29, p = 0.01). Full details can be found in supplementary Table 7.

### Publication bias

Across all injection types, funnel plots generally showed no strong evidence of publication bias (Supplementary material). For TFESI, VAS plots at 1 month, 6 weeks, 3 months, 6 months, and 1 year often had points outside the 95% expected limits, which suggests possible heterogeneity. Egger’s test at 3 months (p = 0.13) was non-significant. Other timepoints could not be tested due to limited study numbers. ODI plots at 1, 3, 6, and 12 months showed moderate asymmetry. Egger’s tests at 3 months (p = 0.17) and 6 months (p = 0.24) were also non-significant. NRS plots at 1 and 3 months were symmetrical, though Egger’s tests were not possible, which limits assessment. For CESI, most plots had too few studies for reliable interpretation. Where Egger’s tests were feasible, no significant small-study effects were found. For IESI, ODI plots at 3 and 6 months showed moderate asymmetry, but Egger’s tests were not possible. Visual inspection suggested that variability was more likely due to heterogeneity in study design and populations rather than systematic publication bias.

## Discussion

Lower back and radicular pain remain a prominent cause of disability and productivity loss, with LDH being a primary contributor to discogenic back pain [5, 31]. Currently, NICE guidelines advocate for a stepwise approach to treatment, beginning with conservative management strategies [42]. First-line interventions include physical therapy, non-steroidal anti-inflammatory drugs (NSAIDs), and patient education. Our meta-analysis, which included 41 studies, demonstrates that ESIs provide significant pain relief and functional improvement across all three examined injection techniques, despite significant heterogeneity between studies and outcome measures. Pooled effect sizes indicate clinically meaningful reductions in pain and disability, particularly within the first 3 to 6 months post-injection. Our findings offer evidence for ESIs as an effective option for patients with persistent symptoms who seek to delay or avoid surgery, as well as those with reversible conditions where temporary symptom control can facilitate rehabilitation and recovery.

However, these findings appear to contrast with the recent clinical practice guideline by Busse et al., which issued strong recommendations against epidural steroid injections for chronic axial and radicular spine pain due to limited evidence of long-term benefit and potential harm [12]. This apparent discrepancy likely reflects fundamental differences in the target populations. Our meta-analysis focuses on patients with symptomatic LDH, a condition with a clearly defined structural pathology. In contrast, the guideline by Busse et al. addresses non-specific, non-cancer-related chronic spine pain, which often lacks radiographic correlates or a discernible pathoanatomic basis. Future research should aim to refine patient selection criteria and develop evidence-based indications for TFESI, CESI, and IESI in the treatment of LDH. Nonetheless, given the inherent limitations of this meta-analysis, we cannot directly establish causality. The contrast underscores persistent uncertainty regarding long-term efficacy and highlights the need for more rigorous studies, clearer clinical indications, and standardized treatment protocols to inform the use of ESIs in practice.

The societal burden of chronic low back pain (CLBP) extends beyond pathology, impacting healthcare costs, lost productivity, and disability compensation. CLBP is a leading cause of years lived with disability and costs the U.S. over $100 billion annually [55, 76, 84]. In Europe, indirect costs from absenteeism often exceed direct medical expenses. ESIs may reduce short-term disability costs and healthcare utilization. A cost-effectiveness analysis found ESIs yield a cost per QALY of £8,975 in outpatient settings, though financial viability depends on reimbursement models [50]. Limited access to ESIs, particularly in rural areas, may increase opioid reliance, exacerbating CLBP's economic impact. ESIs can improve workplace productivity by expediting return-to-work rates, reducing employer disability costs [76]. Additionally, multimodal pain management incorporating ESIs may reduce opioid dependence, enhancing cognitive function and workforce retention [84]. However, financial viability remains linked to healthcare accessibility, regional disparities, and long-term efficacy, requiring policymakers to balance cost savings with recurrence risks and overutilization [84]. Moreover, these projections often rely on assumptions about sustained benefit, which remain uncertain given the variability and short follow-up in much of the existing literature. Without consistent long-term data from controlled trials, cost-effectiveness estimates must be interpreted with caution.

Pain relief, assessed using both the NRS and VAS, was most pronounced in the TFESI group, reinforcing the hypothesis that TFESI provides superior analgesia by directly targeting the affected nerve root. Several RCTs support these findings. Ploumis et al. found TFESI provided significantly better pain relief than CESI at 6 months, particularly for disc herniation [65]. The authors attributed this efficacy to TFESI’s ability to deliver corticosteroids and local anaesthetics precisely at the site of inflammation, reducing nerve root oedema and cytokine-mediated pain transmission [65, 67]. Unlike CESI and IESI, TFESI delivers steroids near the dorsal root ganglion, a highly vascularized structure responsible for nociceptive signal modulation, enabling rapid uptake and prolonged anti-inflammatory effects [74]. However, the lack of placebo controls in most studies RCTs, introduce the placebo effect as a confounder, so the observed benefit may not be attributable solely to the intervention. The widespread use of image guidance in the included studies and the precise localization of TFESI further reduce steroid dispersion and systemic absorption, contributing to longer-lasting pain relief. Tecer et al. found that TFESI was effective regardless of disc herniation type or location, but imaging findings such as high-intensity zones (HIZ) and nerve root impingement (NRI) correlated with improved pain relief outcomes. This suggests that imaging plays a crucial role in improving procedural accuracy and treatment efficacy, which may help explain the superior efficacy of TFESI [79].

CESI, examined in 13 studies, showed steady pain score reductions over time, with notable improvements at 1 and 3 months. Unlike TFESI’s concentrated dose, CESI’s broader corticosteroid spread benefits patients with multi-level pathology or spinal stenosis [39, 53]. However, this diffusion likely contributes to its slower onset compared to TFESI. Higher vascular uptake and systemic absorption may further reduce bioavailability at affected nerve roots [44]. Yet, variation in injection volume, technique, and anatomical targeting across CESI studies makes it difficult to assess the consistency and reproducibility of these outcomes in practice.

IESI, assessed in four studies, provided the least immediate relief but demonstrated a sustained effect. Unlike TFESI’s direct approach, IESI relies on passive steroid diffusion, leading to slower symptom resolution [15, 82]. Epidural fat may act as a reservoir, delaying therapeutic impact [63]. Despite this, IESI benefits patients with bilateral symptoms or central stenosis, where broader steroid spread is advantageous [75]. However, diffusion delays and epidural fibrosis may reduce efficacy, particularly in severe stenosis cases [71]. Additionally, the heterogeneity in follow-up durations within IESI-based studies further restricts conclusions about long-term benefit and recurrence rates.

Functional improvement, measured via the ODI, mirrored pain relief trends, with TFESI yielding the greatest reductions. This suggests that superior pain relief translates to enhanced mobility and rehabilitation participation. Ghahreman et al. reported that TFESI is particularly effective in restoring function in patients with unilateral radiculopathy, as precise corticosteroid delivery to the affected nerve root significantly reduces radicular pain and enhances lower extremity function [26]. Yet across modalities, outcome measurement tools varied in scale and timing, while also being subject to patient interpretation, raising concerns about comparability and clinical significance of reported improvements.

While CESI provides broad corticosteroid distribution, its lack of targeted drug delivery may contribute to a slower resolution of nerve root inflammation. The initial functional improvement is likely due to corticosteroids' broad anti-inflammatory effects, which modulate nociceptive signalling and reduce central sensitization [52]. However, because CESI does not target a specific nerve root, its long-term impact on disability may be less sustained compared to TFESI [82]. This aligns with findings from Manchikanti et al., who noted that CESI is effective for chronic pain relief but does not provide the same robust functional improvements as TFESI [49]. IESI showed gradual functional improvement, likely due to broader but less concentrated steroid dispersion. Unlike TFESI’s targeted relief, IESI provides bilateral drug spread, benefiting patients with central stenosis or broad-based herniations [75]. However, slower diffusion across a larger area may delay symptom resolution, and severe stenosis or epidural fibrosis could further impact efficacy [71].

Given the observed heterogeneity in the literature, and this study, identifying patient-specific predictors of success is crucial for optimising treatment outcomes. Imaging findings, particularly the presence of HIZ, nerve root impingement, and Modic changes on MRI, have been correlated with enhanced pain relief following TFESI, as these structural abnormalities may indicate a more inflammatory pain aetiology, which is more conducive to corticosteroid [20]. Conversely, patients with extensive epidural fibrosis, severe central canal stenosis, or predominantly neuropathic pain mechanisms, as opposed to disc herniation may exhibit limited benefit from ESIs due to reduced drug diffusion or non-inflammatory pain drivers [9]. Additionally, psychological and behavioural factors, including depression, pain catastrophizing, and opioid dependence, have been associated with poorer ESI outcomes, suggesting the need for a multidisciplinary approach in patient selection. Given the wide scope of the above variables, a comprehensive pre-treatment assessment incorporating imaging, clinical history, and psychosocial screening in future studies would enable more granular patient stratification, and more robust sub-group analysis into what factors should be considered when using ESIs in a clinical setting. Future trials should stratify patients accordingly and maintain methodological rigor to allow meaningful subgroup comparisons and guide personalized care.

Our systematic search enabled a comprehensive pooled analysis, but several limitations must be acknowledged. First, there was substantial heterogeneity across studies. This likely reflects differences in patient selection, severity of pathology, injection technique, corticosteroid formulation, and follow-up duration. The absence of standardized protocols and inconsistent use of fluoroscopic guidance may have also impacted procedural accuracy and treatment outcomes. Second, although we conducted a sensitivity analysis including only studies with low risk of bias, we were unable to perform a similar analysis limited to low-risk comparative studies due to the small number of eligible studies. Third, the meta-regression examined study-level variables such as age, sex, body mass index, and number of sessions, but these associations should not be interpreted as individual-level predictors. Inconsistent reporting across studies further limited analysis of the effects of specific corticosteroid types and doses. Variation in the formulations used, including dexamethasone, triamcinolone, and methylprednisolone, complicated direct comparisons. Moreover, the distribution of studies across injection modalities was uneven. TFESI were studied more frequently than IESI or CESI injections. Many studies evaluating IESI and CESI lacked long-term follow-up beyond six months, which limits the strength of conclusions regarding the sustained effectiveness of these approaches. In addition, the majority of included studies lacked appropriate control groups, relying instead on within-group comparisons from baseline to follow-up. This limits interpretability, as such designs are vulnerable to placebo effects, natural recovery, and regression to the mean. In some cases, randomisation procedures were inadequately described or compromised, further weakening causal inference. As a result, definitive treatment efficacy cannot be established without rigorous between-group analyses using well-conducted randomized controlled trials. Another limitation is that SMC calculations for within-group effects required the assumption of a pre–post correlation coefficient, which was not reported in the included studies. We used a conservative estimate (r = 0.5), but the true correlation likely varies between studies. This introduces uncertainty into variance estimates and may affect the precision of pooled effect sizes. Finally, for meta-regression analyses, studies with missing covariate data were excluded from the relevant models, which may have reduced statistical power and could introduce bias if the data were not missing at random.

This meta-analysis suggests that intra-articular injections may offer symptomatic relief in patients with symptomatic LDH, with TFESI demonstrating the largest overall effect size in both pain relief and functional improvement. However, substantial heterogeneity and overlapping indications underscores the need for standardized protocols and further research to enhance generalisability. Future studies should aim for greater methodological consistency and personalized treatment approaches, while also working to establish evidence-based indications for selecting between TFESI, CESI, and IESI to optimize patient outcomes.

## Conflict of interest

The authors of this manuscript declare no relationships with any companies, whose products or services may be related to the subject matter of the article.

## Supplementary Information

Below is the link to the electronic supplementary material.

Supplementary Material 1 (PDF 8.60 MB)
