# Supplementary material for: Intra-articular steroid injections for lumbar disk herniation: a systematic review and meta-analysis
Source: Acta Neurochir (Wien). 2025 Nov 14;167(1):293. doi: 10.1007/s00701-025-06676-4 (PMC12615523; doi:10.1007/s00701-025-06676-4)
Supplement: Supplementary file 1 — Supplementary Material 1 (PDF 8.60 MB) [file 701_2025_6676_MOESM1_ESM.pdf]

Supplementary Material

Supplementary Table 1: Search strategy.

Database	Search terms	Publication dates	Results (n)
Medline	((("lumbar disc herniation".sh. OR "lumbar disc displacement".sh. OR "intervertebral disc displacement".sh. OR "lumbar herniation".tw. OR "lumbar disc disease".tw. OR "herniated disc".tw. OR "slipped disc".tw. OR "disc prolapse".tw. OR "disc protrusion".tw. OR "disc extrusion".tw. OR "disc sequestration".tw. OR "disc rupture".tw. OR "disc derangement".tw. OR "nucleus pulposus herniation".tw. OR "nucleus pulposus prolapse".tw. OR "disc displacement".tw. OR "intervertebral disc herniation".tw. OR "lumbar disc pathology".tw.) AND (("physical therapy".sh. OR "physical therapy modalities".sh. OR "exercise therapy".sh. OR "manual therapy".sh. OR "chiropractic".sh. OR "spinal manipulation".sh. OR "acupuncture".sh. OR "massage therapy".sh. OR "therapeutic exercise".sh. OR "traction".sh. OR "heat therapy".sh. OR "cold therapy".sh. OR "electrotherapy".sh. OR "injection therapy".sh. OR "steroid injections".sh. OR "epidural injections".sh. OR "transforaminal injections".sh. OR "facet joint injections".sh. OR "laser therapy".sh. OR "laser nucleoplasty".sh. OR "yoga".sh. OR "Pilates".sh. OR "physiotherapy".sh. OR "non-surgical treatment".tw. OR "conservative treatment".tw.) OR ("core strengthening".tw. OR "core stability".tw. OR "stretching exercises".tw. OR "aerobic exercise".tw. OR "mobilization".tw. OR "osteopathy".tw. OR "neural mobilization".tw. OR "ergonomic education".tw. OR "posture education".tw. OR "transcutaneous electrical nerve stimulation".tw. OR "TENS".tw. OR "dry needling".tw. OR "back school".tw. OR "mechanical traction".tw. OR "postural training".tw.))))	1948 - 2024	n = 8,359
Embase	((("lumbar disc herniation".sh. OR "lumbar disc displacement".sh. OR "intervertebral disc displacement".sh. OR "lumbar herniation".tw. OR "lumbar disc disease".tw. OR "herniated disc".tw. OR "slipped disc".tw. OR "disc prolapse".tw. OR "disc protrusion".tw. OR "disc extrusion".tw. OR "disc sequestration".tw. OR "disc rupture".tw. OR "disc derangement".tw. OR "nucleus pulposus herniation".tw. OR "nucleus pulposus prolapse".tw. OR "disc displacement".tw. OR "intervertebral disc herniation".tw. OR "lumbar disc pathology".tw.) AND (("physical therapy".sh. OR "physical therapy modalities".sh. OR "exercise therapy".sh. OR "manual therapy".sh. OR "chiropractic".sh. OR "spinal manipulation".sh. OR "acupuncture".sh. OR "massage therapy".sh. OR "therapeutic exercise".sh. OR "traction".sh. OR "heat therapy".sh. OR "cold therapy".sh. OR "electrotherapy".sh. OR "injection therapy".sh. OR	1947 - 2024	n = 4,870

	"steroid injections".sh. OR "epidural injections".sh. OR "transforaminal injections".sh. OR "facet joint injections".sh. OR "laser therapy".sh. OR "laser nucleoplasty".sh. OR "yoga".sh. OR "Pilates".sh. OR "physiotherapy".sh. OR "non-surgical treatment".tw. OR "conservative treatment".tw.) OR ("core strengthening".tw. OR "core stability".tw. OR "stretching exercises".tw. OR "aerobic exercise".tw. OR "mobilization".tw. OR "osteopathy".tw. OR "neural mobilization".tw. OR "ergonomic education".tw. OR "posture education".tw. OR "transcutaneous electrical nerve stimulation".tw. OR "TENS".tw. OR "dry needling".tw. OR "back school".tw. OR "mechanical traction".tw. OR "postural training".tw.))))		
Scopus	TITLE-ABS-KEY ((("lumbar disc herniation" OR "lumbar disc displacement" OR "intervertebral disc displacement" OR "lumbar herniation" OR "lumbar disc disease" OR "herniated disc" OR "slipped disc" OR "disc prolapse" OR "disc protrusion" OR "disc extrusion" OR "disc sequestration" OR "disc rupture" OR "disc derangement" OR "nucleus pulposus herniation" OR "nucleus pulposus prolapse" OR "disc displacement" OR "intervertebral disc herniation" OR "lumbar disc pathology") AND (("physical therapy" OR "physical therapy modalities" OR "exercise therapy" OR "manual therapy" OR "chiropractic" OR "spinal manipulation" OR "acupuncture" OR "massage therapy" OR "therapeutic exercise" OR "traction" OR "heat therapy" OR "cold therapy" OR "electrotherapy" OR "injection therapy" OR "steroid injections" OR "epidural injections" OR "transforaminal injections" OR "facet joint injections" OR "laser therapy" OR "laser nucleoplasty" OR "yoga" OR "Pilates" OR "physiotherapy" OR "non-surgical treatment" OR "conservative treatment") OR ("core strengthening" OR "core stability" OR "stretching exercises" OR "aerobic exercise" OR "mobilization" OR "osteopathy" OR "neural mobilization" OR "ergonomic education" OR "posture education" OR "transcutaneous electrical nerve stimulation" OR "TENS" OR "dry needling" OR "back school" OR "mechanical traction" OR "postural training"))))	1943 - 2024	n = 6,190
Pubmed	((("lumbar disc herniation"[tiab] OR "lumbar disc displacement"[tiab] OR "intervertebral disc displacement"[MeSH] OR "lumbar herniation"[tiab] OR "lumbar disc disease"[tiab] OR "herniated disc"[tiab] OR "slipped disc"[tiab] OR "disc prolapse"[tiab] OR "disc protrusion"[tiab] OR "disc extrusion"[tiab] OR "disc sequestration"[tiab] OR "disc rupture"[tiab] OR "disc derangement"[tiab] OR "nucleus pulposus herniation"[tiab] OR "nucleus pulposus prolapse"[tiab] OR "disc displacement"[tiab] OR "intervertebral disc herniation"[tiab] OR "lumbar disc pathology"[tiab])) AND (("physical therapy"[tiab] OR "physical therapy modalities"[MeSH] OR "exercise therapy"[MeSH] OR "manual therapy"[tiab]	1943 - 2024	n = 8,724

	OR "chiropractic"[MeSH] OR "spinal manipulation"[tiab] OR "acupuncture"[MeSH] OR "massage therapy"[tiab] OR "therapeutic exercise"[tiab] OR "traction"[MeSH] OR "heat therapy"[tiab] OR "cold therapy"[tiab] OR "electrotherapy"[tiab] OR "injection therapy"[tiab] OR "steroid injections"[tiab] OR "epidural injections"[tiab] OR "transforaminal injections"[tiab] OR "facet joint injections"[tiab] OR "laser therapy"[MeSH] OR "laser nucleoplasty"[tiab] OR "yoga"[MeSH] OR "Pilates"[tiab] OR "physiotherapy"[tiab] OR "non-surgical treatment"[tiab] OR "conservative treatment"[tiab]) OR ("core strengthening"[tiab] OR "core stability"[tiab] OR "stretching exercises"[tiab] OR "aerobic exercise"[tiab] OR "mobilization"[tiab] OR "osteopathy"[tiab] OR "neural mobilization"[tiab] OR "ergonomic education"[tiab] OR "posture education"[tiab] OR "transcutaneous electrical nerve stimulation"[tiab] OR "TENS"[tiab] OR "dry needling"[tiab] OR "back school"[tiab] OR "mechanical traction"[tiab] OR "postural training"[tiab])))		

Supplementary table 1 describes the search strings used for each database.

Supplementary Table 2: Inclusion and exclusion criteria.

**Inclusion criteria**	**Exclusion criteria**
● Published in the English language<br>● Peer-reviewed journals<br>● Adults (aged 18 years and older) of any gender with symptomatic lumbar disc herniation, confirmed by MRI or CT scans, representing the primary diagnosis.<br>● .Studies exploring a modality of epidural steroid injections for LDH	● All non-English languages<br>● Commentaries, case reports, narrative reviews, letters to editors, books<br>● Any animal studies and lab-based studies<br>● Studies on children and adolescents (<18 years)<br>● Studies where lumbar disc herniation diagnosis is based solely on myelography.<br>● Disc herniations of areas of the spine other than the lumbar region<br>● Injections of substances other than steroids into the epidural space

In Supplementary Table 2, the inclusion and exclusion criteria used when filtering studies based off search results are shown.

Supplementary Table 3: ROB-2

Title	Author (Year)	Intervention vs Comparator	Primary Outcome(s)	Randomisation Bias	Deviations from Interventions	Missing Data Bias	Measurement Bias	Selection Bias	Overall Risk of Bias
CT-guided Pulsed Radiofrequency Combined with Steroid Injection for Sciatica from Herniated Disk: A Randomized Trial.	Napoli et al. (2023)	PRF + TFESI vs TFESI alone	Pain Scores (NRS), Functional Status (RMDQ, ODI)	Low	Low	Low	Some Concerns	Some Concerns	Low
Ultrasound-Guided Transforaminal Injections of Platelet-Rich Plasma Compared with Steroid in Lumbar Disc Herniation: A Prospective, Randomized, Controlled Study.	Xu et al. (2021)	PRP vs Steroid Injection	Pain Scores (VAS, PPTs), Functional Status (ODI, SF-36)	Low	Some Concerns	Low	Some Concerns	Low	Some Concerns
"Platelet-Rich Plasma" epidural injection an emerging strategy in lumbar disc herniation: a Randomized Controlled Trial.	Wongjarupong et al. (2023)	PRP vs Steroid Injection	Pain Scores (VAS), Functional Status (ODI), Adverse Events, Treatment Failure	Low	Some Concerns	Low	Some Concerns	Low	Some Concerns
Comparative effectiveness of lumbar transforaminal epidural steroid injections with particulate versus nonparticulate corticosteroids for lumbar radicular pain due to intervertebral disc herniation: a	Kennedy et al. (2014)	Particulate vs Non-particulate Steroid Injection	Pain Scores (NRS), Functional Status (ODI),	Low	Low	Low	Some Concerns	Low	Low

prospective, randomized, double-blind trial.			Surgical Rates, Number of Injections						
Effect of fluoroscopically guided caudal epidural steroid or local anesthetic injections in the treatment of lumbar disc herniation and radiculitis: a randomized, controlled, double blind trial with a two-year follow-up.	Manchikanti et al. (2012)	Steroid vs Non-Steroid Injection	Pain Scores (NRS), Functional Status (ODI)	Low	Some Concerns	Low	Some Concerns	Low	Low
Caudal epidural steroid injection versus transforaminal ESI for unilateral S1 radiculopathy: a prospective, randomized trial.	Ozturk et al. (2023)	Epidural steroid injection vs Sham	Pain Scores (NRS), Functional Status (ODI)	Some Concerns	Low	Low	Some Concerns	Low	Some Concerns
The role of fluoroscopic interlaminar epidural injections in managing chronic pain of lumbar disc herniation or radiculitis: a randomized, double-blind trial.	Manchikanti et al. (2013)	Transforaminal epidural steroid injection vs PRF	Pain Scores (NRS), Functional Status (ODI)	Low	Low	Some Concerns	Some Concerns	Low	Low
Lumbar retrodiscal versus post-ganglionic transforaminal epidural steroid injection for the treatment of lumbar intervertebral disc herniations.	Park et al. (2011)	Epidural steroid injection vs PRP	Pain Scores (VAS, Roland 5-point Score), Patient Satisfaction Index	Low	Some Concerns	Low	Some Concerns	Some Concerns	Some Concerns
Epidural corticosteroid injections for sciatica due to herniated nucleus pulposus.	Carette et al. (1997)	Epidural Corticosteroid vs Placebo	Functional Status (ODI), Surgery Rates	Low	Low	Low	Some Concerns	Some Concerns	Some Concerns

Transforaminal epidural injections in chronic lumbar disc herniation: a randomized, double-blind, active-control trial.	Manchikanti et al. (2014)	Transforaminal Epidural Injection vs Placebo	Pain Scores (NRS), Functional Status (ODI)	Some Concerns	Low	Low	Some Concerns	Low	Some Concerns
Comparison of the effectiveness of lumbar transforaminal epidural injection with particulate and nonparticulate corticosteroids in lumbar radiating pain.	Park et al. (2010)	Particulate vs Non-particulate Steroid Injection	Pain Scores (VAS, McGill Pain Questionnaire), Functional Status (ODI)	Some Concerns	Some Concerns	Low	Some Concerns	Some Concerns	Some Concerns
Microdiscectomy compared with transforaminal epidural steroid injection for persistent radicular pain caused by prolapsed intervertebral disc: the NERVES RCT.	Wilby et al. (2021)	Microdiscectomy vs TFESI	Pain Scores (VAS), Functional Status (ODI, RMDQ)	Low	Low	Low	Some Concerns	Some Concerns	Low
Effectiveness of Epidural Steroid Injection Depending on Discoradicular Contact: A Prospective Randomized Trial	Budrovac et al. (2023)	Discoradicular Contact vs No Discoradicular Contact	Pain Scores (VAS), Functional Status (ODI)	Low	Some Concerns	Low	Some Concerns	Some Concerns	Some Concerns
Transforaminal epidural steroid injection combined with radio frequency for the treatment of lumbar disc herniation: a 2-year follow-up	Wei at al. (2021)	TFESI + RF vs TFESI	Pain Scores (VAS), Functional Status (ODI), GPE Scale	Low	Low	Low	Some Concerns	Some Concerns	Low

A randomized, double-blind, active-control trial of the effectiveness of lumbar interlaminar epidural injections in disc herniation.	Manchikanti et al. (2014)	ESI vs Local Anesthetic	Pain Scores (NRS), Functional Status (ODI)	Low	Low	Low	Some Concerns	Low	Low
Caudal epidural steroid injection ultrasound-guided versus fluoroscopy-guided in treatment of refractory lumbar disc prolapse with radiculopathy	Elashmawy et al. (2021)	Ultrasound vs Fluoroscopy-guided Epidural Steroid Injection	Pain Scores (VAS), Functional Status (ODI)	Low	Low	Low	Some Concerns	Some Concerns	Low
Long-Term comparative study between transforaminal and interlaminar epidural injection of steroids in lumbar radiculopathy due to single-level disc herniation	Soliman et al. (2018)	TFESI vs ILESI	Pain Scores (VAS), Functional Status (ODI)	Some Concerns	Some Concerns	Low	Some Concerns	Some Concerns	Some Concerns
Efficacy of Caudal Epidural Steroid Injection with Targeted Indwelling Catheter and Manipulation in Managing Patients with Lumbar Disk Herniation and Radiculopathy: A Prospective, Randomized, Single-Blind Controlled Trial	Yin et al. (2018)	Epidural Steroid Injection vs Manipulation	Pain Scores (VAS), Functional Status (ODI)	Low	Some Concerns	Low	Some Concerns	Low	Low
Selective nerve root blocks vs. caudal epidural injection for single level prolapsed lumbar intervertebral disc – A prospective randomized study	Singh et al. (2017)	Selective Nerve Root Block vs CESI	Pain Scores (VAS), Functional Status (ODI)	Low	Low	Low	Some Concerns	Low	Low
Outcome of single level disc prolapse treated with transforaminal steroid versus epidural steroid versus caudal steroids	Kamble et al. (2016)	TFESI vs ILESI vs CESI	Pain Scores (VAS), Functional Status (ODI)	Low	Low	Low	Some Concerns	Some Concerns	Low

RMDQ - Roland Morris Disability Questionnaire
PPTs - Pressure Pain Thresholds
GPE - Global Perceived Effect

Supplementary Table 3 describes RoB-2 analyses of all (20) RCTs included in the study.

Supplementary Table 4: ROBIN-I

**Title**	**Author (Year)**	**Bias due to Confounding**	**Bias in Selection of Participants**	**Bias in Classification of Interventions**	**Bias due to Deviations from Intended Interventions**	**Bias due to Missing Data**	**Bias in Measurement of Outcomes**	**Bias in Selection of Reported Outcomes**	**Overall Risk of Bias**
Comparison of treatment outcomes in lumbar disc herniation patients treated with epidural steroid injections: interlaminar versus transforaminal approach.	Bensler et al. (2020)	Moderate	Moderate	Low	Moderate	Moderate	Serious	Moderate	Moderate
Transforaminal Epidural Steroid Injection in the Treatment of Pain in Foraminal and Paramedian Lumbar Disc Herniations.	Guclu et al. (2020)	Serious	Moderate	Low	Moderate	Low	Serious	Low	Moderate
Evaluation of the effectiveness of transforaminal epidural steroid injection in far lateral lumbar disc herniations.	Evran et al. (2021)	Serious	Moderate	Low	Moderate	Low	Serious	Moderate	Serious
Transforaminal Epidural Steroid Injection Improves Neuropathic Pain in Lumbar Radiculopathy: A Prospective, Clinical Study.	Sencan et al. (2021)	Serious	Moderate	Low	Moderate	Low	Serious	Moderate	Serious
309 patients treated with fluoroscopy-guided caudal epidural injection for lumbar disc herniation.	Akşan et al. (2022)	Moderate	Moderate	Low	Moderate	Moderate	Serious	Moderate	Moderate
Optimal Timing and Outcome of Transforaminal Epidural Steroid Injection for the Management of Radicular Pain due to Extruded Lumbar Disc Herniation.	Guclu et al. (2023)	Moderate	Moderate	Low	Moderate	Moderate	Serious	Moderate	Moderate

Comparison of the Effect of Single Lumbar Transforaminal Epidural Steroid Injections for the Treatment of L4-5 and L5-S1 Paramedian Disc Herniation.	Adilay et al. (2019)	Serious	Moderate	Low	Moderate	Moderate	Serious	Moderate	Serious
Fluoroscopically guided caudal epidural steroid injections for axial low back pain associated with central disc protrusions: a prospective outcome study.	Lee et al. (2019)	Serious	Moderate	Low	Moderate	Moderate	Serious	Moderate	Serious
Short-term assessment of periradicular corticosteroid injections in lumbar radiculopathy associated with disc pathology.	Viton et al. (1998)	Serious	Moderate	Low	Moderate	Low	Serious	Moderate	Serious
Effectiveness of epidural steroid injection for the management of symptomatic herniated lumbar disc.	Baral et al. (2011)	Moderate	Serious	Low	Moderate	Moderate	Serious	Moderate	Serious
The Outcome of Epidural Injections in Lumbar Radiculopathy Is Not Dependent on the Presence of Disc Herniation on Magnetic Resonance Imaging: Assessment of Short-Term and Long-Term Efficacy.	Verheijen et al. (2021)	Moderate	Moderate	Low	Moderate	Moderate	Serious	Serious	Serious
Caudal epidural steroid injection for chronic low back pain: A prospective analysis of 107 patients.	Dernek et al. (2022)	Serious	Moderate	Low	Moderate	Moderate	Serious	Moderate	Serious
Epidural steroid injection in patients with lumbosacral radiculopathy in Abuja, Nigeria.	Kawu et al. (2012)	Serious	Serious	Low	Moderate	Serious	Serious	Serious	Serious
Comparison of Epidural Steroid Injection Efficiency with Two Different Doses in Radiculopathies Associated with Lumbar Disc Herniation.	Ozsoy-Unubol et al. (2018)	Serious	Moderate	Low	Moderate	Moderate	Serious	Moderate	Serious
The Synergistic Effect of Combined Transforaminal and Caudal Epidural Steroid Injection in Recurrent Lumbar Disc Herniations.	Evran et al. (2021)	Serious	Serious	Low	Moderate	Serious	Serious	Serious	Serious

Functional Outcomes and Successful Predictors of Lumbar Transforaminal Epidural Steroid Injections (LTFESIs) for Lumbar Radiculopathy Under Fluoroscopic Guidance: A Prospective Study.	Dhandapani et al. (2023)	Serious	Moderate	Low	Moderate	Moderate	Serious	Moderate	Serious
Transforaminal Epidural Injection for Far Lateral Lumbar Disc Herniations: An Alternative to Surgery or Just a Delay?	Serifoglu et al. (2024)	Moderate	Moderate	Low	Moderate	Moderate	Serious	Moderate	Serious
Comparison of the Particulate Steroids, Betamethasone and Methylprednisolone, in Caudal Steroid Injection Under Ultrasound Guidance	Guler at al. (2023)	Serious	Moderate	Low	Moderate	Low	Serious	Moderate	Serious
Comparison Between a Single Subpedicular Transforaminal Epidural Steroid Injection and Lateral Recess Steroid Injection in Reducing Paracentral Disc Herniation–Related Chronic Neuropathic Leg Pain: A Retrospective Study	Jain et al. (2021)	Moderate	Moderate	Low	Moderate	Moderate	Serious	Moderate	Serious
THE EFFICACY OF TRANSFORAMINAL EPIDURAL STEROID INJECTION (TFESI) IN SINGLE LEVEL LUMBAR DISC HERNIATION	Evran et al. (2019)	Moderate	Moderate	Low	Moderate	Moderate	Serious	Moderate	Serious
Transforaminal epidural steroid injection combined with pulsed radio frequency on spinal nerve root for the treatment of lumbar disc herniation	Ding et al (2018)	Moderate	Moderate	Low	Moderate	Moderate	Serious	Serious	Serious

Supplementary Table 4 describes ROBIN-1 analyses of all non-RCTs (21) included in the study.

Supplementary Table 5: OCEBM Levels of Evidence

Title	Author (Year)	Study Design	Level of Evidence
Comparison of treatment outcomes in lumbar disc herniation patients treated with epidural steroid injections: interlaminar versus transforaminal approach.	Bensler et al. (2020)	Retrospective Cohort	3
CT-guided Pulsed Radiofrequency Combined with Steroid Injection for Sciatica from Herniated Disk: A Randomized Trial.	Napoli et al. (2023)	RCT	2
Ultrasound-Guided Transforaminal Injections of Platelet-Rich Plasma Compared with Steroid in Lumbar Disc Herniation: A Prospective, Randomized, Controlled Study.	Xu et al. (2021)	RCT	2
"Platelet-Rich Plasma" epidural injection an emerging strategy in lumbar disc herniation: a Randomized Controlled Trial.	Wongjarupong et al. (2023)	RCT	2
Transforaminal Epidural Steroid Injection in the Treatment of Pain in Foraminal and Paramedian Lumbar Disc Herniations.	Guclu et al. (2020)	Retrospective Cohort	3
Evaluation of the effectiveness of transforaminal epidural steroid injection in far lateral lumbar disc herniations.	Evran et al. (2021)	Retrospective Cohort	3
Comparative effectiveness of lumbar transforaminal epidural steroid injections with particulate versus nonparticulate corticosteroids for lumbar radicular pain due to intervertebral disc herniation: a prospective, randomized, double-blind trial.	Kennedy et al. (2014)	RCT	2
Effect of fluoroscopically guided caudal epidural steroid or local anesthetic injections in the treatment of lumbar disc herniation and radiculitis: a randomized, controlled, double blind trial with a two-year follow-up.	Manchikanti et al. (2012)	RCT	2
Transforaminal Epidural Steroid Injection Improves Neuropathic Pain in Lumbar Radiculopathy: A Prospective, Clinical Study.	Sencan et al. (2021)	Prospective Cohort	3
309 patients treated with fluoroscopy-guided caudal epidural injection for lumbar disc herniation.	Akşan et al. (2022)	Prospective Cohort	3
Optimal Timing and Outcome of Transforaminal Epidural Steroid Injection for the Management of Radicular Pain due to Extruded Lumbar Disc Herniation.	Guclu et al. (2023)	Prospective Cohort	3
Caudal epidural steroid injection versus transforaminal ESI for unilateral S1 radiculopathy: a prospective, randomized trial.	Ozturk et al. (2023)	RCT	2

Comparison of the Effect of Single Lumbar Transforaminal Epidural Steroid Injections for the Treatment of L4-5 and L5-S1 Paramedian Disc Herniation.	Adilay et al. (2019)	Retrospective Cohort	3
The role of fluoroscopic interlaminar epidural injections in managing chronic pain of lumbar disc herniation or radiculitis: a randomized, double-blind trial.	Manchikanti et al. (2013)	RCT	2
Fluoroscopically guided caudal epidural steroid injections for axial low back pain associated with central disc protrusions: a prospective outcome study.	Lee et al. (2019)	Prospective Cohort	3
Lumbar retrodiscal versus post-ganglionic transforaminal epidural steroid injection for the treatment of lumbar intervertebral disc herniations.	Park et al. (2011)	RCT	2
Epidural corticosteroid injections for sciatica due to herniated nucleus pulposus.	Carette et al. (1997)	RCT	2
Short-term assessment of periradicular corticosteroid injections in lumbar radiculopathy associated with disc pathology.	Viton et al. (1998)	Prospective Cohort	3
Effectiveness of epidural steroid injection for the management of symptomatic herniated lumbar disc.	Baral et al. (2011)	Prospective Cohort	3
Transforaminal epidural injections in chronic lumbar disc herniation: a randomized, double-blind, active-control trial.	Manchikanti et al. (2014)	RCT	2
The Outcome of Epidural Injections in Lumbar Radiculopathy Is Not Dependent on the Presence of Disc Herniation on Magnetic Resonance Imaging: Assessment of Short-Term and Long-Term Efficacy.	Verheijen et al. (2021)	Retrospective Cohort	3
Caudal epidural steroid injection for chronic low back pain: A prospective analysis of 107 patients.	Dernek et al. (2022)	Retrospective Cohort	3
Comparison of the effectiveness of lumbar transforaminal epidural injection with particulate and nonparticulate corticosteroids in lumbar radiating pain.	Park et al. (2010)	RCT	2
Epidural steroid injection in patients with lumbosacral radiculopathy in Abuja, Nigeria.	Kawu et al. (2012)	Prospective Cohort	3
Comparison of Epidural Steroid Injection Efficiency with Two Different Doses in Radiculopathies Associated with Lumbar Disc Herniation.	Ozsoy-Unubol et al. (2018)	Prospective Cohort	3
The Synergistic Effect of Combined Transforaminal and Caudal Epidural Steroid Injection in Recurrent Lumbar Disc Herniations.	Evran et al. (2021)	Prospective Cohort	3

Functional Outcomes and Successful Predictors of Lumbar Transforaminal Epidural Steroid Injections (LTFESIs) for Lumbar Radiculopathy Under Fluoroscopic Guidance: A Prospective Study.	Dhandapani et al. (2023)	Prospective Cohort	3
Transforaminal Epidural Injection for Far Lateral Lumbar Disc Herniations: An Alternative to Surgery or Just a Delay?	Serifoglu et al. (2024)	Retrospecti ve Cohort	3
Microdiscectomy compared with transforaminal epidural steroid injection for persistent radicular pain caused by prolapsed intervertebral disc: the NERVES RCT.	Wilby et al. (2021)	RCT	2
Comparison of the Particulate Steroids, Betamethasone and Methylprednisolone, in Caudal Steroid Injection Under Ultrasound Guidance	Guler at al. (2023)	Retrospecti ve Cohort	3
Effectiveness of Epidural Steroid Injection Depending on Discoradicular Contact: A Prospective Randomized Trial	Budrovac et al. (2023)	RCT	2
Transforaminal epidural steroid injection combined with radio frequency for the treatment of lumbar disc herniation: a 2-year follow-up	Wei at al. (2021)	RCT	2
Comparison Between a Single Subpedicular Transforaminal Epidural Steroid Injection and Lateral Recess Steroid Injection in Reducing Paracentral Disc Herniation–Related Chronic Neuropathic Leg Pain: A Retrospective Study	Jain et al. (2021)	Retrospecti ve Cohort	3
A randomized, double-blind, active-control trial of the effectiveness of lumbar interlaminar epidural injections in disc herniation.	Manchikanti et al. (2014)	RCT	2
Caudal epidural steroid injection ultrasound-guided versus fluoroscopy-guided in treatment of refractory lumbar disc prolapse with radiculopathy	Elashmawy et al. (2021)	RCT	2
THE EFFICACY OF TRANSFORAMINAL EPIDURAL STEROID INJECTION (TFESI) IN SINGLE LEVEL LUMBAR DISC HERNIATION	Evran et al. (2019)	Retrospecti ve Cohort	3
Long-Term comparative study between transforaminal and interlaminar epidural injection of steroids in lumbar radiculopathy due to single-level disc herniation	Soliman et al. (2018)	RCT	2
Efficacy of Caudal Epidural Steroid Injection with Targeted Indwelling Catheter and Manipulation in Managing Patients with Lumbar Disk Herniation and Radiculopathy: A Prospective, Randomized, Single-Blind Controlled Trial	Yin et al. (2018)	RCT	2
Transforaminal epidural steroid injection combined with pulsed radio frequency on spinal nerve root for the treatment of lumbar disc herniation	Ding et al (2018)	Retrospecti ve Cohort	3
Selective nerve root blocks vs. caudal epidural injection for single level prolapsed lumbar intervertebral disc – A prospective randomized study	Singh et al. (2017)	RCT	2

Outcome of single level disc prolapse treated with transforaminal steroid versus epidural steroid versus caudal steroids	Kamble et al. (2016)	RCT	2

Supplementary Table 5 describes the OCEBM Levels of Evidence for all included studies.

Supplementary Table 6: GRADE Assessment

Title	Author (Year)	Study Design	GRADE
Comparison of treatment outcomes in lumbar disc herniation patients treated with epidural steroid injections: interlaminar versus transforaminal approach.	Bensler et al. (2020)	Retrospective Cohort	Moderate
CT-guided Pulsed Radiofrequency Combined with Steroid Injection for Sciatica from Herniated Disk: A Randomized Trial.	Napoli et al. (2023)	RCT	High
Ultrasound-Guided Transforaminal Injections of Platelet-Rich Plasma Compared with Steroid in Lumbar Disc Herniation: A Prospective, Randomized, Controlled Study.	Xu et al. (2021)	RCT	Moderate
"Platelet-Rich Plasma" epidural injection an emerging strategy in lumbar disc herniation: a Randomized Controlled Trial.	Wongjarupong et al. (2023)	RCT	Moderate
Transforaminal Epidural Steroid Injection in the Treatment of Pain in Foraminal and Paramedian Lumbar Disc Herniations.	Guclu et al. (2020)	Retrospective Cohort	Moderate
Evaluation of the effectiveness of transforaminal epidural steroid injection in far lateral lumbar disc herniations.	Evran et al. (2021)	Retrospective Cohort	Low
Comparative effectiveness of lumbar transforaminal epidural steroid injections with particulate versus nonparticulate corticosteroids for lumbar radicular pain due to intervertebral disc herniation: a prospective, randomized, double-blind trial.	Kennedy et al. (2014)	RCT	Moderate
Effect of fluoroscopically guided caudal epidural steroid or local anesthetic injections in the treatment of lumbar disc herniation and radiculitis: a randomized, controlled, double blind trial with a two-year follow-up.	Manchikanti et al. (2012)	RCT	High
Transforaminal Epidural Steroid Injection Improves Neuropathic Pain in Lumbar Radiculopathy: A Prospective, Clinical Study.	Sencan et al. (2021)	Prospective Cohort	Low
309 patients treated with fluoroscopy-guided caudal epidural injection for lumbar disc herniation.	Akşan et al. (2022)	Prospective Cohort	Low
Optimal Timing and Outcome of Transforaminal Epidural Steroid Injection for the Management of Radicular Pain due to Extruded Lumbar Disc Herniation.	Guclu et al. (2023)	Prospective Cohort	Moderate
Caudal epidural steroid injection versus transforaminal ESI for unilateral S1 radiculopathy: a prospective, randomized trial.	Ozturk et al. (2023)	RCT	Moderate
Comparison of the Effect of Single Lumbar Transforaminal Epidural Steroid Injections for the Treatment of L4-5 and L5-S1 Paramedian Disc Herniation.	Adilay et al. (2019)	Retrospective Cohort	Low

The role of fluoroscopic interlaminar epidural injections in managing chronic pain of lumbar disc herniation or radiculitis: a randomized, double-blind trial.	Manchikanti et al. (2013)	RCT	High
Fluoroscopically guided caudal epidural steroid injections for axial low back pain associated with central disc protrusions: a prospective outcome study.	Lee et al. (2019)	Prospective Cohort	Low
Lumbar retrodiscal versus post-ganglionic transforaminal epidural steroid injection for the treatment of lumbar intervertebral disc herniations.	Park et al. (2011)	RCT	Moderate
Epidural corticosteroid injections for sciatica due to herniated nucleus pulposus.	Carette et al. (1997)	RCT	Moderate
Short-term assessment of periradicular corticosteroid injections in lumbar radiculopathy associated with disc pathology.	Viton et al. (1998)	Prospective Cohort	Low
Effectiveness of epidural steroid injection for the management of symptomatic herniated lumbar disc.	Baral et al. (2011)	Prospective Cohort	Low
Transforaminal epidural injections in chronic lumbar disc herniation: a randomized, double-blind, active-control trial.	Manchikanti et al. (2014)	RCT	Moderate
The Outcome of Epidural Injections in Lumbar Radiculopathy Is Not Dependent on the Presence of Disc Herniation on Magnetic Resonance Imaging: Assessment of Short-Term and Long-Term Efficacy.	Verheijen et al. (2021)	Retrospective Cohort	Moderate
Caudal epidural steroid injection for chronic low back pain: A prospective analysis of 107 patients.	Dernek et al. (2022)	Retrospective Cohort	Low
Comparison of the effectiveness of lumbar transforaminal epidural injection with particulate and nonparticulate corticosteroids in lumbar radiating pain.	Park et al. (2010)	RCT	Moderate
Epidural steroid injection in patients with lumbosacral radiculopathy in Abuja, Nigeria.	Kawu et al. (2012)	Prospective Cohort	Moderate
Comparison of Epidural Steroid Injection Efficiency with Two Different Doses in Radiculopathies Associated with Lumbar Disc Herniation.	Ozsoy-Unubol et al. (2018)	Prospective Cohort	Low
The Synergistic Effect of Combined Transforaminal and Caudal Epidural Steroid Injection in Recurrent Lumbar Disc Herniations.	Evran et al. (2021)	Prospective Cohort	Low
Functional Outcomes and Successful Predictors of Lumbar Transforaminal Epidural Steroid Injections (LTFESIs) for Lumbar Radiculopathy Under Fluoroscopic Guidance: A Prospective Study.	Dhandapani et al. (2023)	Prospective Cohort	Low

Transforaminal Epidural Injection for Far Lateral Lumbar Disc Herniations: An Alternative to Surgery or Just a Delay?	Serifoglu et al. (2024)	Retrospective Cohort	Low
Microdiscectomy compared with transforaminal epidural steroid injection for persistent radicular pain caused by prolapsed intervertebral disc: the NERVES RCT.	Wilby et al. (2021)	RCT	Moderate
Comparison of the Particulate Steroids, Betamethasone and Methylprednisolone, in Caudal Steroid Injection Under Ultrasound Guidance	Guler at al. (2023)	Retrospective Cohort	Low
Effectiveness of Epidural Steroid Injection Depending on Discoradicular Contact: A Prospective Randomized Trial	Budrovac et al. (2023)	RCT	Moderate
Transforaminal epidural steroid injection combined with radio frequency for the treatment of lumbar disc herniation: a 2-year follow-up	Wei at al. (2021)	RCT	Moderate
Comparison Between a Single Subpedicular Transforaminal Epidural Steroid Injection and Lateral Recess Steroid Injection in Reducing Paracentral Disc Herniation–Related Chronic Neuropathic Leg Pain: A Retrospective Study	Jain et al. (2021)	Retrospective Cohort	Low
A randomized, double-blind, active-control trial of the effectiveness of lumbar interlaminar epidural injections in disc herniation.	Manchikanti et al. (2014)	RCT	High
Caudal epidural steroid injection ultrasound-guided versus fluoroscopy-guided in treatment of refractory lumbar disc prolapse with radiculopathy	Elashmawy et al. (2021)	RCT	High
THE EFFICACY OF TRANSFORAMINAL EPIDURAL STEROID INJECTION (TFESI) IN SINGLE LEVEL LUMBAR DISC HERNIATION	Evran et al. (2019)	Retrospective Cohort	Low
Long-Term comparative study between transforaminal and interlaminar epidural injection of steroids in lumbar radiculopathy due to single-level disc herniation	Soliman et al. (2018)	RCT	Moderate
Efficacy of Caudal Epidural Steroid Injection with Targeted Indwelling Catheter and Manipulation in Managing Patients with Lumbar Disk Herniation and Radiculopathy: A Prospective, Randomized, Single-Blind Controlled Trial	Yin et al. (2018)	RCT	Moderate
Transforaminal epidural steroid injection combined with pulsed radio frequency on spinal nerve root for the treatment of lumbar disc herniation	Ding et al (2018)	Retrospective Cohort	Low
Selective nerve root blocks vs. caudal epidural injection for single level prolapsed lumbar intervertebral disc – A prospective randomized study	Singh et al. (2017)	RCT	Moderate
Outcome of single level disc prolapse treated with transforaminal steroid versus epidural steroid versus caudal steroids	Kamble et al. (2016)	RCT	High

Supplementary Table 6 describes the GRADE Assessment of all included studies.

Supplementary table 7: Meta-regression								
**Domain**	**Injection**	**Covariate**	**Model**	**Beta (95% CIs)**	**SE**	**p**	**I2**	**Tau2**
NRS	CESI	Age	RE	-0.96 (-1.52 to -0.41)	0.28	$p < 0.01$	$I^2 = 99.28\%$	$\tau^2 = 0.70$
NRS	CESI	Followup	RE	-0.02 (-0.13 to 0.08)	0.05	$p = 0.64$	$I^2 = 99.62\%$	$\tau^2 = 1.59$
NRS	CESI	Male %	RE	5.80 (0.42 to 11.17)	2.74	$p = 0.03$	$I^2 = 99.54\%$	$\tau^2 = 1.08$
NRS	CESI	Sample Size	RE	0.06 (0.01 to 0.10)	0.02	$p = 0.01$	$I^2 = 99.47\%$	$\tau^2 = 0.95$
NRS	IESI	Age	FE	-0.35 (-0.40 to -0.29)	0.03	$p < 0.01$	$I^2 < 0.01\%$	$\tau^2 < 0.01$
NRS	IESI	Followup	RE	-0.03 (-0.11 to 0.04)	0.04	$p = 0.34$	$I^2 = 96.80\%$	$\tau^2 = 0.61$
NRS	IESI	Male %	FE	-16.51 (-19.15 to -13.88)	1.34	$p < 0.01$	$I^2 < 0.01\%$	$\tau^2 < 0.01$
NRS	IESI	Sample Size	FE	0.06 (0.05 to 0.07)	0.01	$p < 0.01$	$I^2 < 0.01\%$	$\tau^2 < 0.01$
NRS	TFESI	Age	RE	-0.06 (-0.16 to 0.04)	0.05	$p = 0.22$	$I^2 = 99.51\%$	$\tau^2 = 2.71$
NRS	TFESI	BMI	RE	0.17 (-0.28 to 0.61)	0.23	$p = 0.46$	$I^2 = 99.67\%$	$\tau^2 = 2.64$
NRS	TFESI	Followup	RE	-0.21 (-0.33 to -0.09)	0.06	$p < 0.01$	$I^2 = 98.96\%$	$\tau^2 = 1.48$
NRS	TFESI	Male %	RE	-11.94 (-28.58 to 4.70)	8.49	$p = 0.16$	$I^2 = 99.50\%$	$\tau^2 = 2.68$
NRS	TFESI	Sample Size	RE	0.00 (-0.02 to 0.01)	0.01	$p = 0.60$	$I^2 = 99.56\%$	$\tau^2 = 3.02$
ODI	CESI	Age	RE	-0.19 (-1.06 to 0.68)	0.44	$p = 0.66$	$I^2 = 99.87\%$	$\tau^2 = 102.68$
ODI	CESI	BMI	RE	-3.23 (-4.37 to -2.09)	0.58	$p < 0.01$	$I^2 = 58.69\%$	$\tau^2 = 5.76$
ODI	CESI	Followup	RE	0.34 (-0.32 to 1.01)	0.34	$p = 0.31$	$I^2 = 99.81\%$	$\tau^2 = 88.52$
ODI	CESI	Male %	RE	-20.51 (-60.66 to 19.63)	20.48	$p = 0.32$	$I^2 = 98.64\%$	$\tau^2 = 80.81$
ODI	CESI	Sample Size	RE	-0.02 (-0.22 to 0.17)	0.10	$p = 0.81$	$I^2 = 99.85\%$	$\tau^2 = 92.86$
ODI	IESI	Age	FE	-0.51 (-1.29 to 0.27)	0.40	$p = 0.20$	$I^2 = 0.13\%$	$\tau^2 < 0.01$
ODI	IESI	Followup	FE	-0.04 (-0.10 to 0.02)	0.03	$p = 0.16$	$I^2 = 0.02\%$	$\tau^2 < 0.01$
ODI	IESI	Male %	FE	8.89 (-37.69 to 55.46)	23.76	$p = 0.71$	$I^2 = 0.11\%$	$\tau^2 < 0.01$
ODI	IESI	Sample Size	FE	-0.04 (-0.08 to 0.00)	0.02	$p = 0.06$	$I^2 = 0.06\%$	$\tau^2 < 0.01$
ODI	TFESI	Age	RE	-0.99 (-1.35 to -0.62)	0.18	$p < 0.01$	$I^2 = 99.08\%$	$\tau^2 = 110.96$
ODI	TFESI	BMI	RE	11.69 (7.67 to 15.70)	2.05	$p < 0.01$	$I^2 = 98.96\%$	$\tau^2 = 82.24$
ODI	TFESI	Followup	RE	-0.39 (-1.10 to 0.32)	0.36	$p = 0.28$	$I^2 = 99.48\%$	$\tau^2 = 173.69$
ODI	TFESI	Male %	RE	-20.51 (-52.47 to 11.45)	16.31	$p = 0.21$	$I^2 = 99.46\%$	$\tau^2 = 175.44$
ODI	TFESI	Sample Size	RE	-0.02 (-0.07 to 0.03)	0.03	$p = 0.46$	$I^2 = 99.43\%$	$\tau^2 = 175.86$

VAS	CESI	Age	RE	-1.46 (-2.63 to -0.29)	0.60	$p = 0.01$	$I^2 = 99.99\%$	$\tau^2 = 275.92$
VAS	CESI	BMI	FE	0.19 (0.13 to 0.25)	0.03	$p < 0.01$	$I^2 < 0.01\%$	$\tau^2 < 0.01$
VAS	CESI	Followup	RE	-1.04 (-3.17 to 1.08)	1.09	$p = 0.34$	$I^2 = 99.99\%$	$\tau^2 = 332.63$
VAS	CESI	Male %	RE	37.84 (-73.06 to 148.74)	56.58	$p = 0.50$	$I^2 = 99.99\%$	$\tau^2 = 508.49$
VAS	CESI	Sample Size	RE	-0.20 (-0.23 to -0.18)	0.01	$p < 0.01$	$I^2 = 99.85\%$	$\tau^2 = 24.11$
VAS	IESI	Age	FE	0.01 (-0.17 to 0.19)	0.09	$p = 0.91$	$I^2 < 0.01\%$	$\tau^2 < 0.01$
VAS	IESI	Followup	FE	-0.01 (-0.10 to 0.07)	0.04	$p = 0.77$	$I^2 = 19.54\%$	$\tau^2 < 0.01$
VAS	IESI	Male %	FE	-2.06 (-37.80 to 33.69)	18.24	$p = 0.91$	$I^2 < 0.01\%$	$\tau^2 < 0.01$
VAS	IESI	Sample Size	FE	0.01 (-0.01 to 0.03)	0.01	$p = 0.46$	$I^2 = 0.15\%$	$\tau^2 < 0.01$
VAS	TFESI	Age	RE	0.30 (-0.68 to 1.27)	0.50	$p = 0.55$	$I^2 = 100.00\%$	$\tau^2 = 584.33$
VAS	TFESI	BMI	RE	-28.42 (-32.73 to -24.12)	2.20	$p < 0.01$	$I^2 = 93.92\%$	$\tau^2 = 0.24$
VAS	TFESI	Followup	RE	0.10 (-1.78 to 1.97)	0.96	$p = 0.92$	$I^2 = 99.99\%$	$\tau^2 = 517.87$
VAS	TFESI	Male %	RE	-111.00 (-176.01 to -46.00)	33.17	$p < 0.01$	$I^2 = 99.99\%$	$\tau^2 = 417.10$
VAS	TFESI	Sample Size	RE	-0.03 (-0.06 to 0.00)	0.02	$p = 0.07$	$I^2 = 99.99\%$	$\tau^2 = 472.60$
Supplementary Table 7 displays the results of the meta-regression. RE = Random Effects; FE = Fixed Effects; SE = Standard Error								

Supplementary Figures

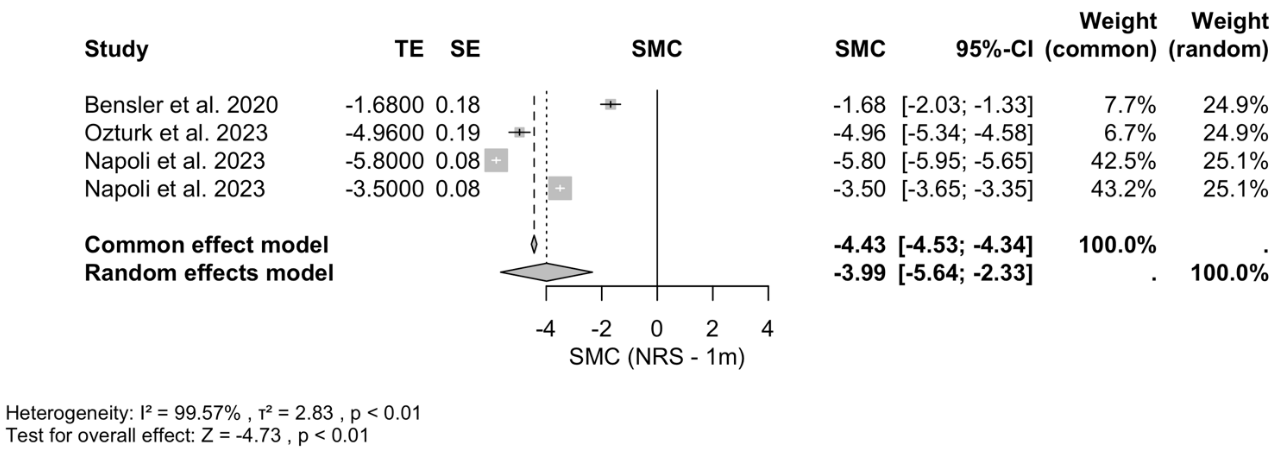

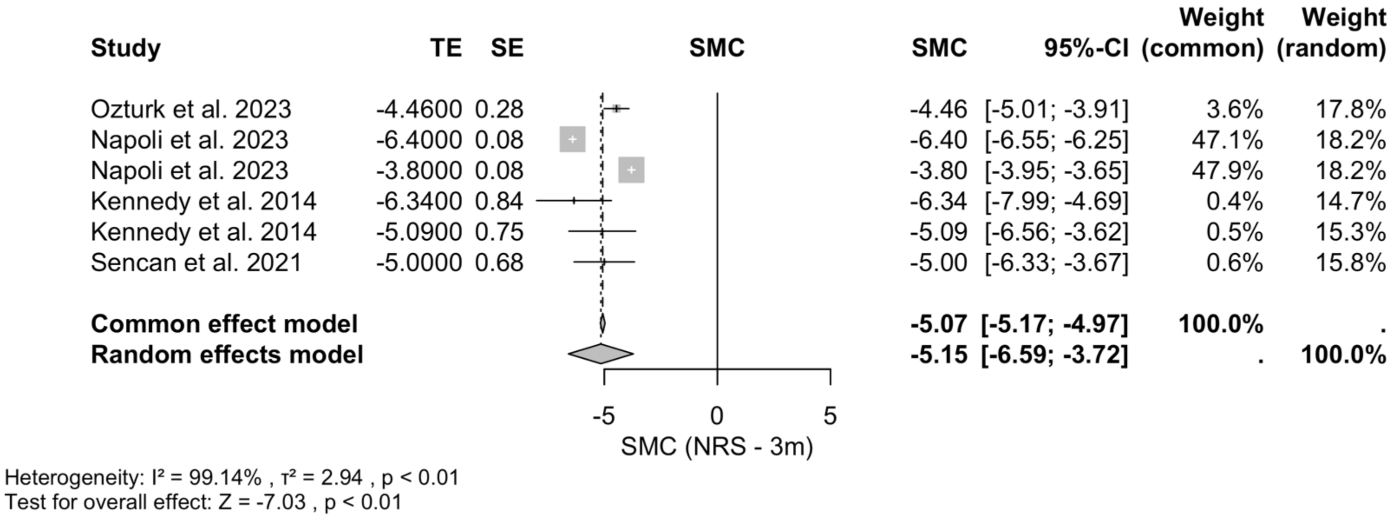

Supplementary Figure 1: Forest Plot for NRS in TFESI Comparing Baseline to specified timeframes

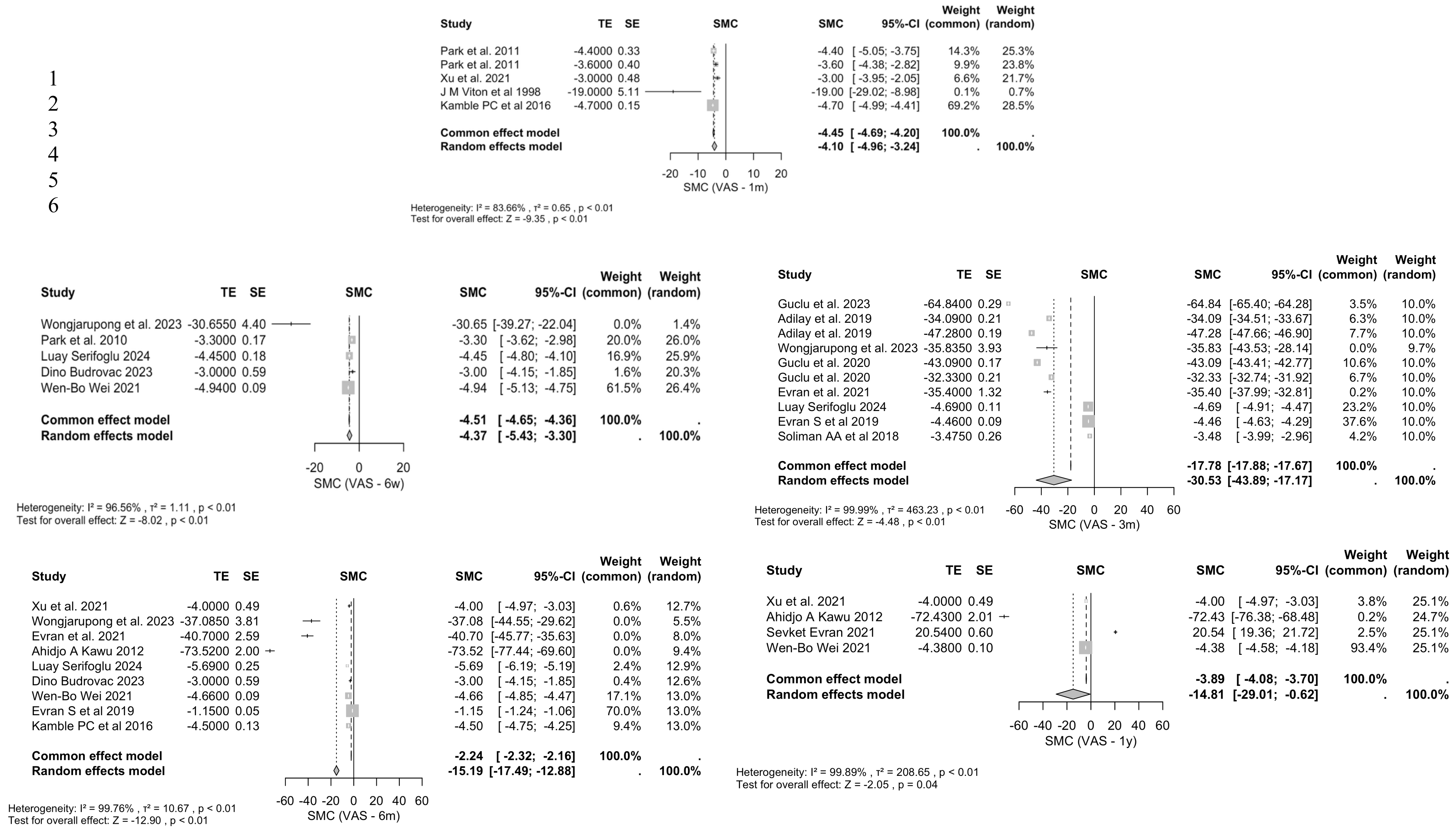

Supplementary Figure 2: Forest Plot for VAS in TFESI Comparing Baseline to specified timeframes

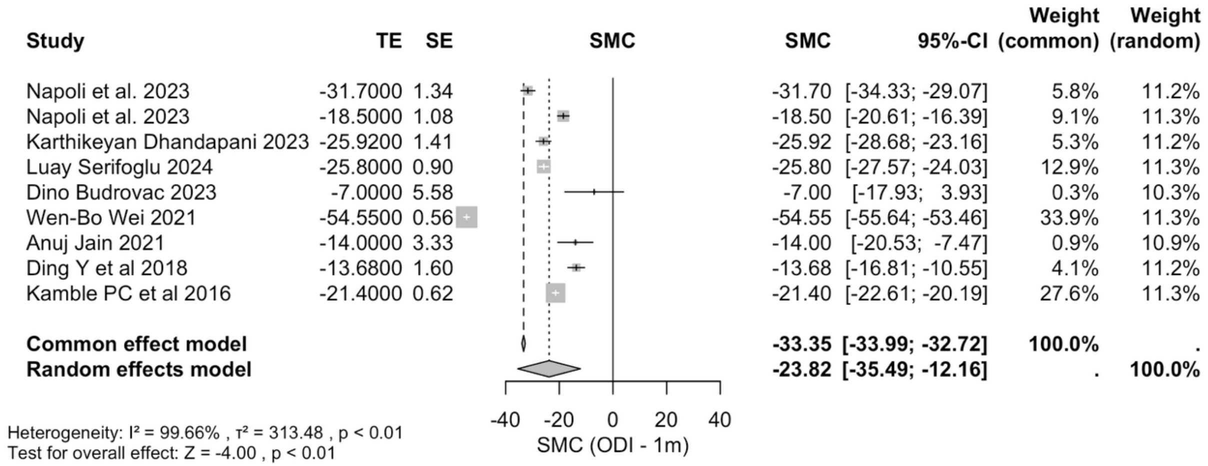

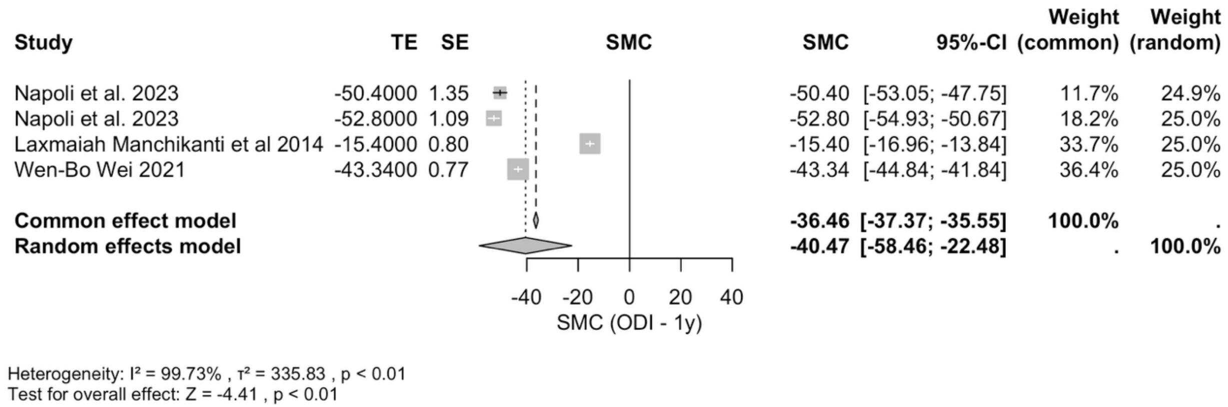

Supplementary Figure 3: Forest Plot for ODI in TFESI Comparing Baseline to specified timeframes

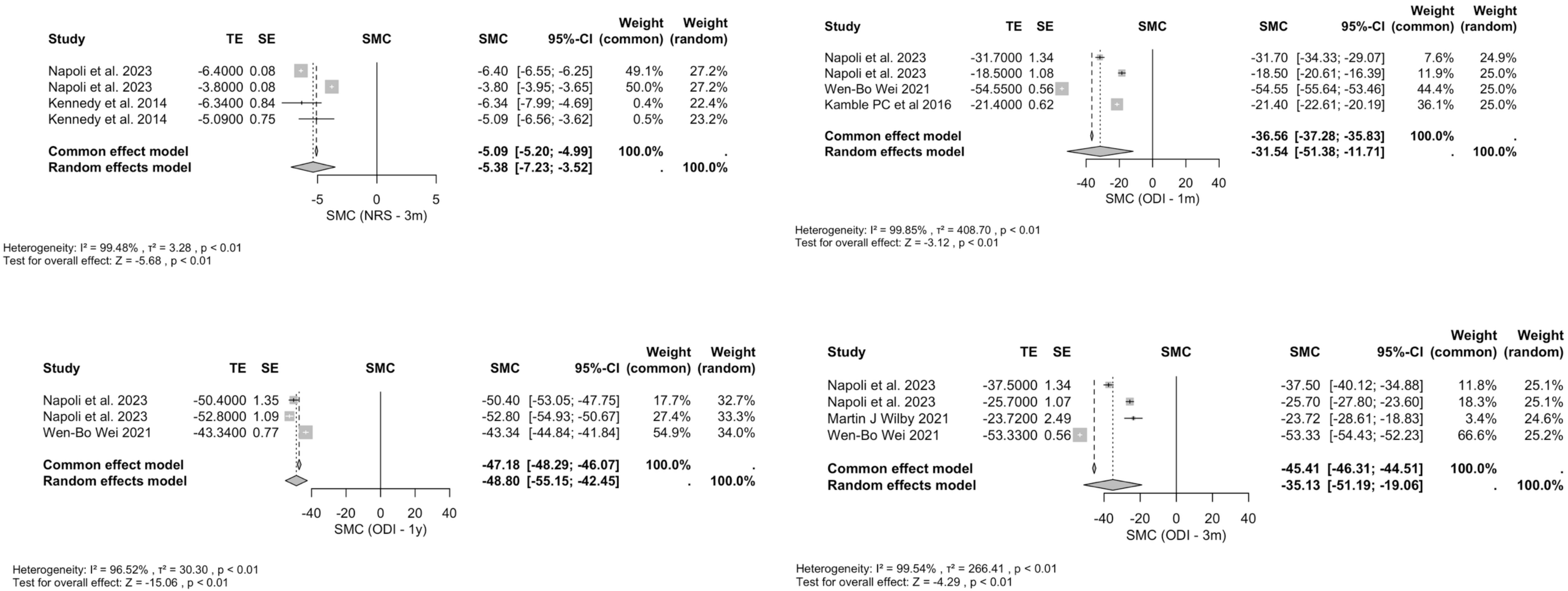

Supplementary Figure 4: Forest Plots for the low risk of bias TFESI sensitivity analysis, comparing Baseline to various time points

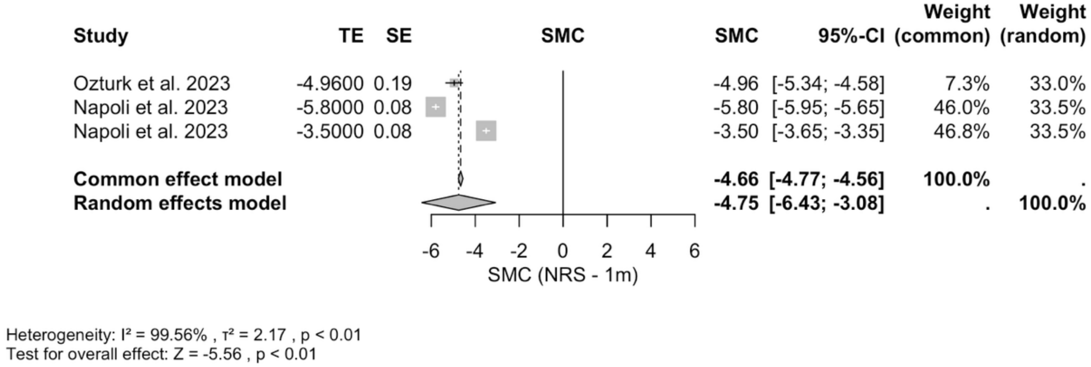

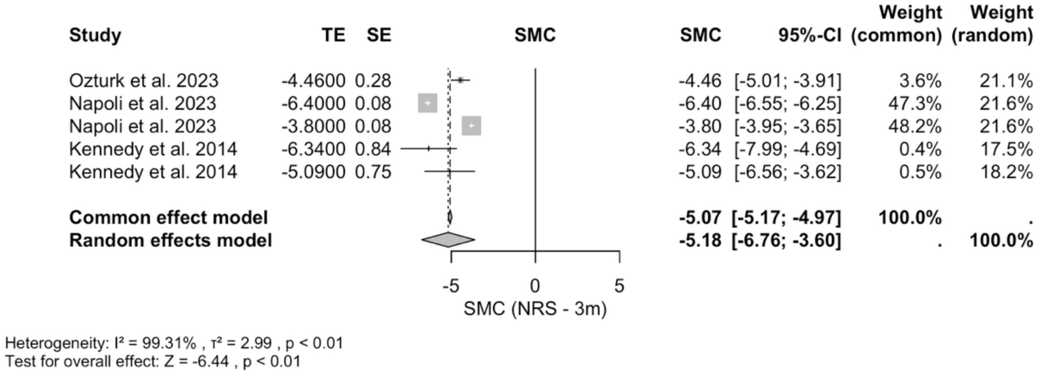

Supplementary Figure 5: NRS Forest Plots for the randomised controlled trials only TFESI sensitivity analysis, comparing Baseline to various time points

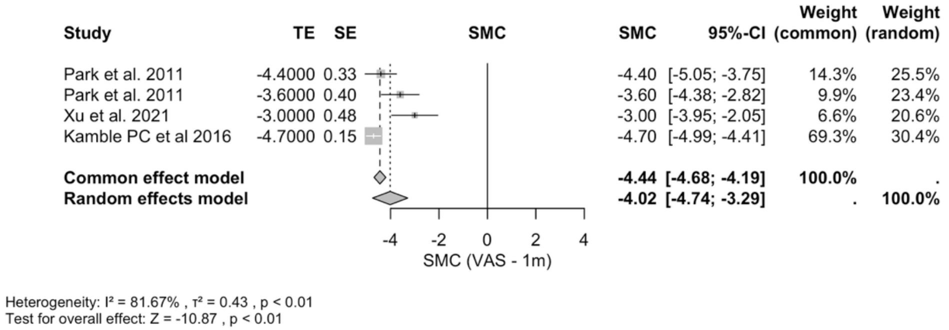

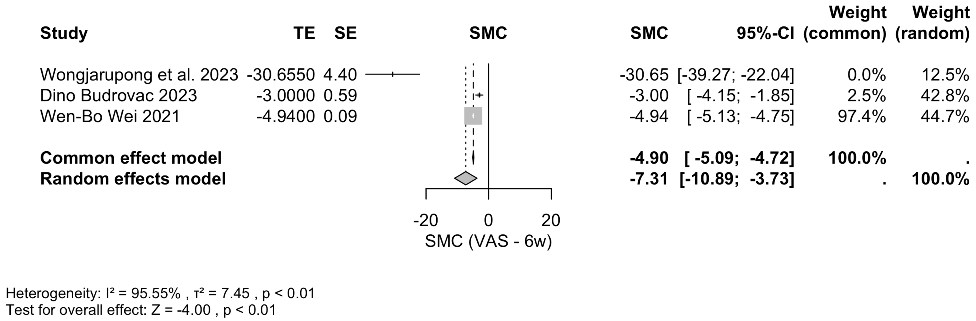

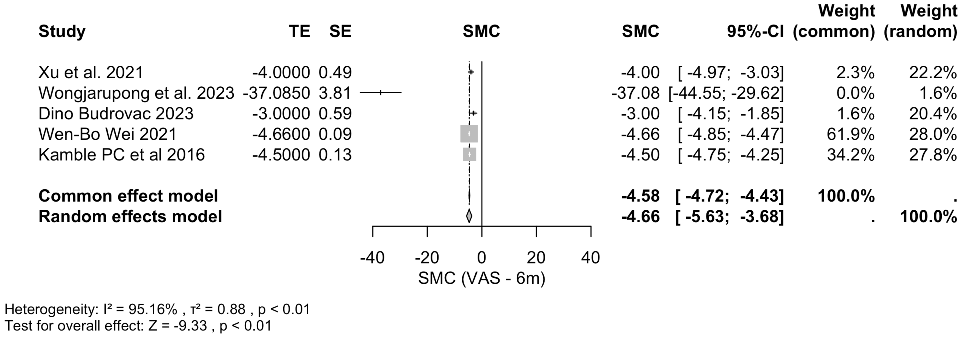

Supplementary Figure 6: VAS Forest Plots for the randomised controlled trials only TFESI sensitivity analysis, comparing Baseline to various time points

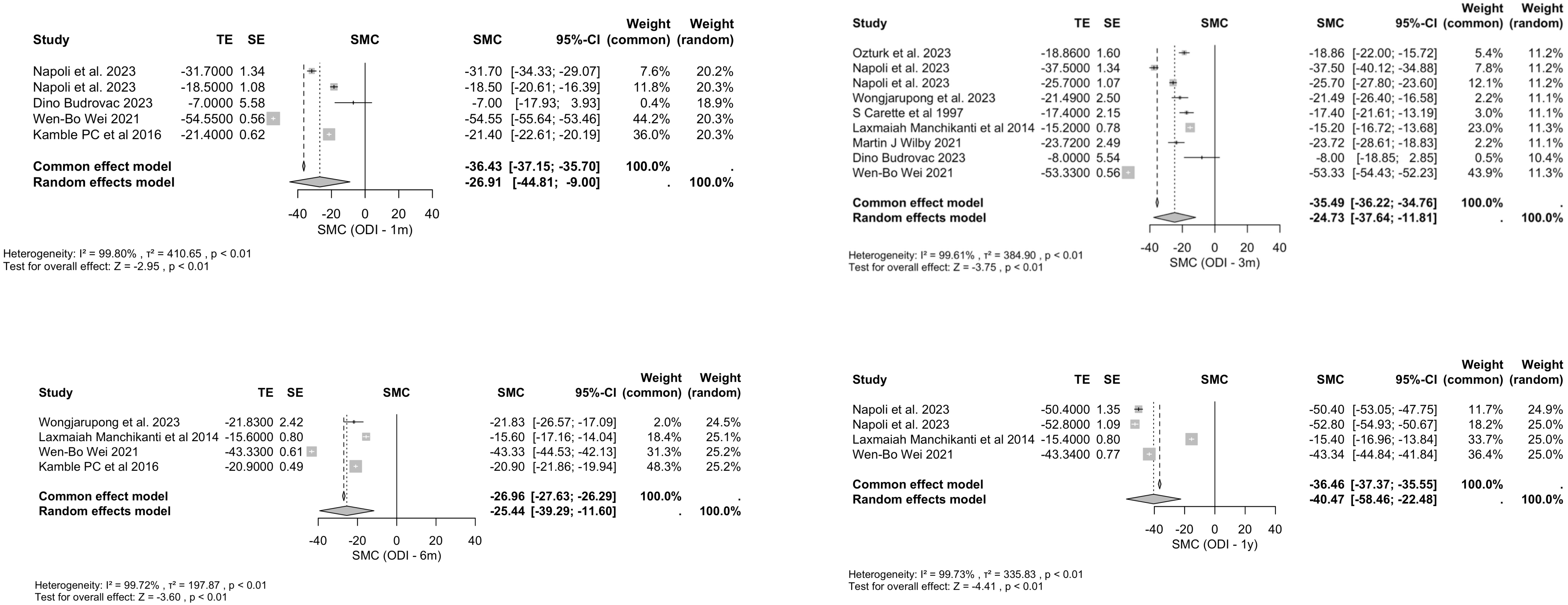

Supplementary Figure 7: ODI Forest Plots for the randomised controlled trials only TFESI sensitivity analysis, comparing Baseline to various time points

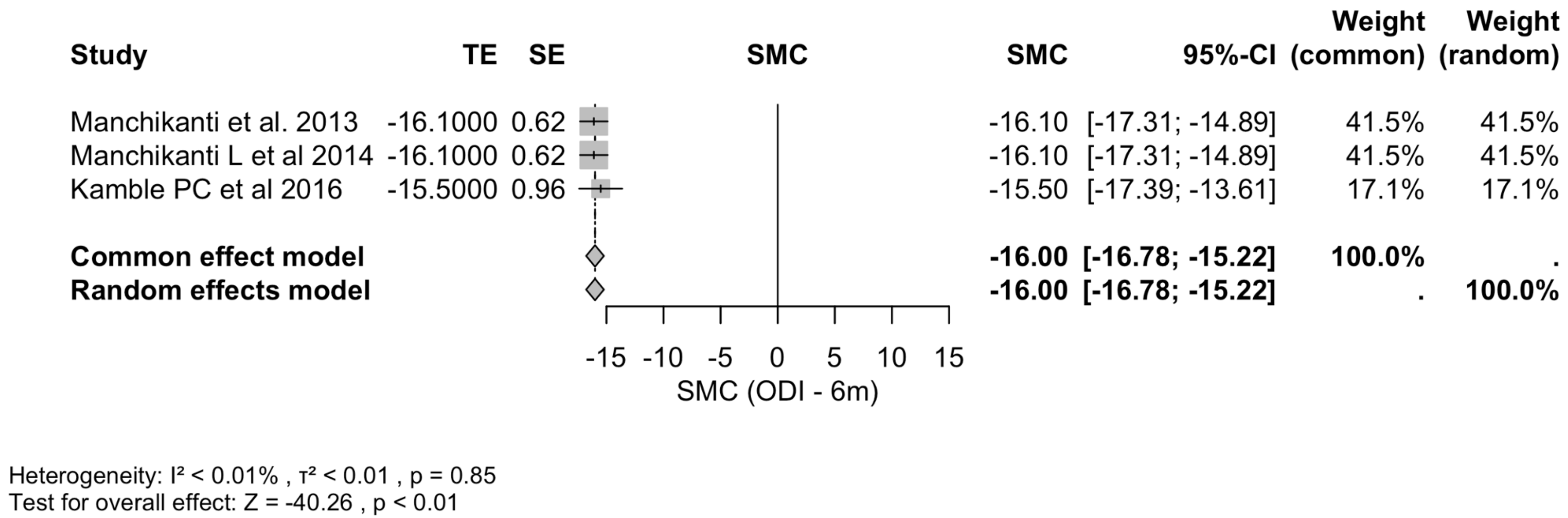

Supplementary Figure 8: ODI Forest Plots for the low risk of bias IESI sensitivity analysis, comparing Baseline to 6 months

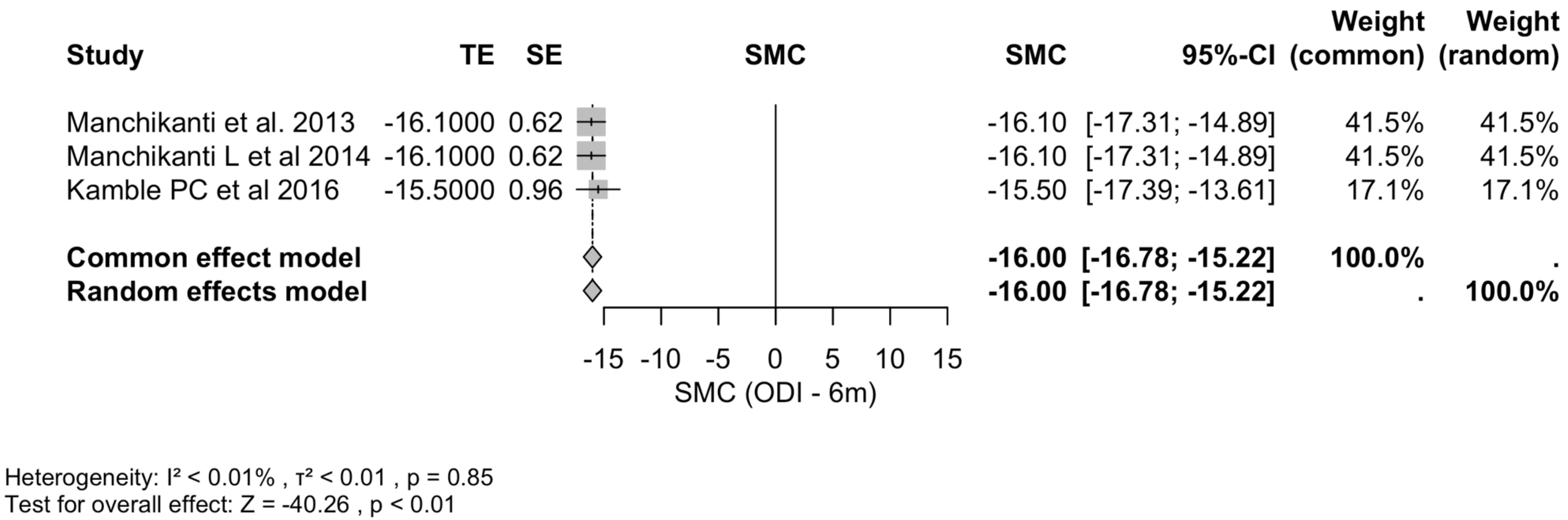

Supplementary Figure 9: ODI Forest Plots for the randomised controlled trials only IESI sensitivity analysis, comparing Baseline to 6 months

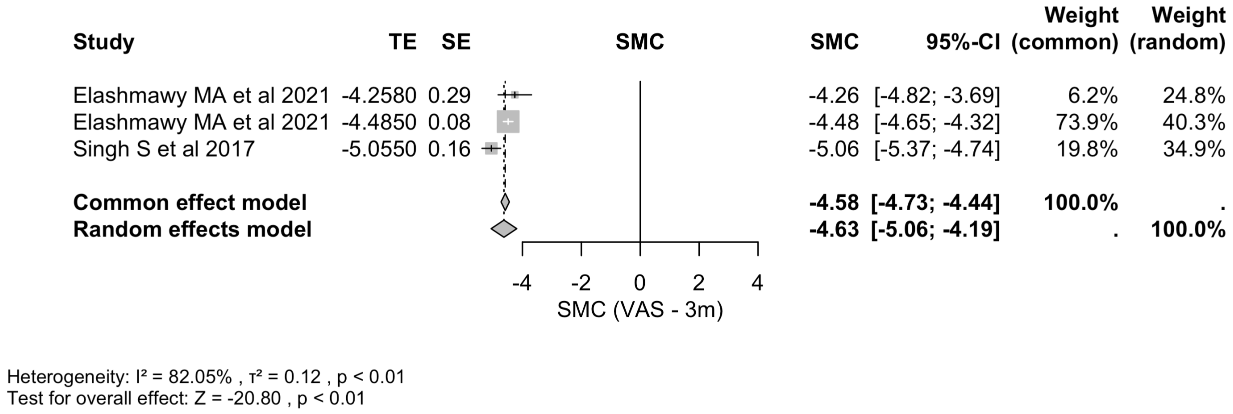

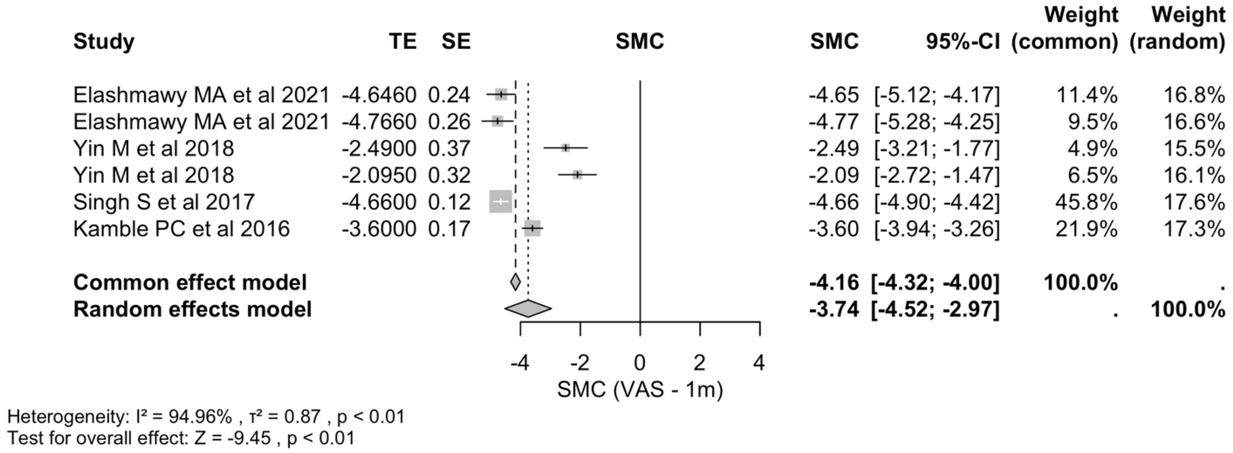

Supplementary Figure 10: VAS Forest Plots for the low risk of bias only CESI sensitivity analysis, comparing Baseline to various timeframes

Study	TE	SE	SMC	95%-CI	Weight (common)	Weight (random)
Elashmawy MA et al 2021	-34.4400	1.83	-34.44	[-38.03; -30.85]	0.3%	16.4%
Elashmawy MA et al 2021	-33.6900	1.86	-33.69	[-37.34; -30.04]	0.3%	16.4%
Yin M et al 2018	-12.1400	1.53	-12.14	[-15.13; -9.15]	0.4%	16.6%
Yin M et al 2018	-8.7400	1.22	-8.74	[-11.14; -6.34]	0.7%	16.7%
Singh S et al 2017	-4.5700	0.10	-4.57	[-4.77; -4.37]	96.3%	16.9%
Kamble PC et al 2016	-15.9000	0.73	-15.90	[-17.32; -14.48]	2.0%	16.9%
**Common effect model**			**-5.03**	**[-5.23; -4.84]**	**100.0%**	**.**
**Random effects model**			**-18.13**	**[-26.57; -9.69]**	**.**	**100.0%**

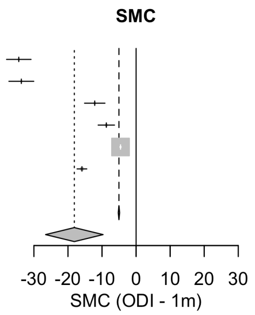

Heterogeneity: $I^2 = 99.35\%$, $\tau^2 = 109.48$, $p < 0.01$
Test for overall effect: $Z = -4.21$, $p < 0.01$

Study	TE	SE	SMC	95%-CI	Weight (common)	Weight (random)
Manchikanti et al. 2012	-14.3000	0.75	-14.30	[-15.78; -12.82]	4.5%	25.6%
Elashmawy MA et al 2021	-31.8200	2.31	-31.82	[-36.34; -27.30]	0.5%	24.4%
Elashmawy MA et al 2021	-29.9100	2.37	-29.91	[-34.56; -25.26]	0.5%	24.3%
Singh S et al 2017	-4.4200	0.16	-4.42	[-4.74; -4.10]	94.6%	25.7%
**Common effect model**			**-5.11**	**[-5.42; -4.80]**	**100.0%**	**.**
**Random effects model**			**-19.84**	**[-29.79; -9.89]**	**.**	**100.0%**

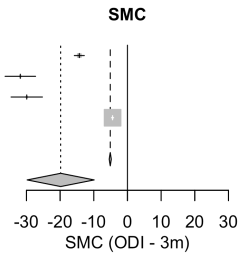

Heterogeneity: $I^2 = 99.27\%$, $\tau^2 = 100.27$, $p < 0.01$
Test for overall effect: $Z = -3.91$, $p < 0.01$

Study	TE	SE	SMC	95%-CI	Weight (common)	Weight (random)
Manchikanti et al. 2012	-14.2000	0.80	-14.20	[-15.77; -12.63]	4.3%	33.2%
Singh S et al 2017	-4.4900	0.18	-4.49	[-4.84; -4.14]	87.1%	33.5%
Kamble PC et al 2016	-15.9000	0.57	-15.90	[-17.01; -14.79]	8.6%	33.3%
**Common effect model**			**-5.89**	**[-6.22; -5.57]**	**100.0%**	**.**
**Random effects model**			**-11.51**	**[-20.08; -2.94]**	**.**	**100.0%**

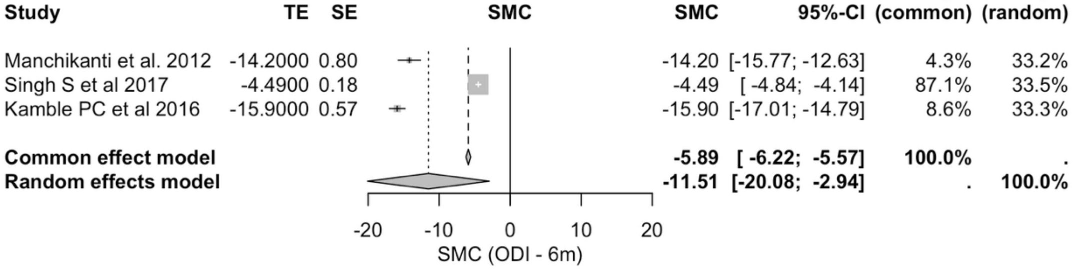

Heterogeneity: $I^2 = 99.58\%$, $\tau^2 = 57.02$, $p < 0.01$
Test for overall effect: $Z = -2.63$, $p < 0.01$

Supplementary Figure 11: ODI Forest Plots for the low risk of bias only CESI sensitivity analysis, comparing Baseline to various timeframes

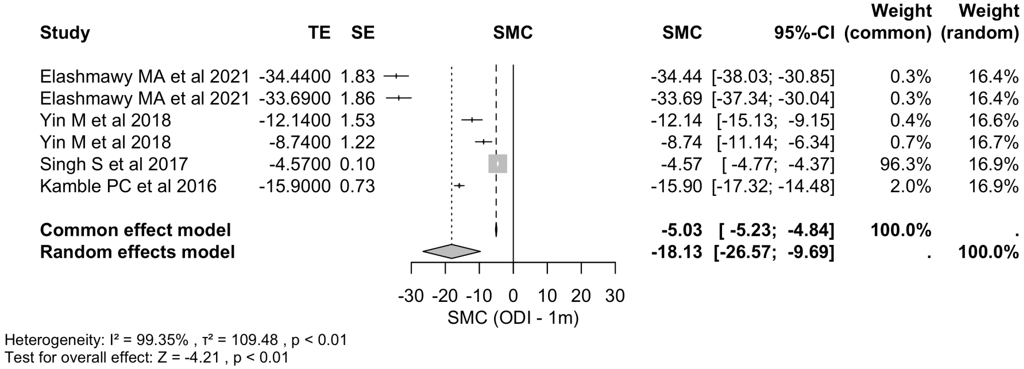

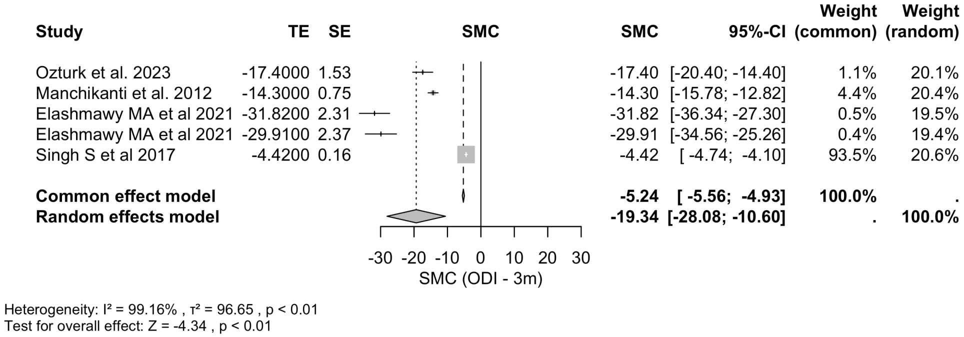

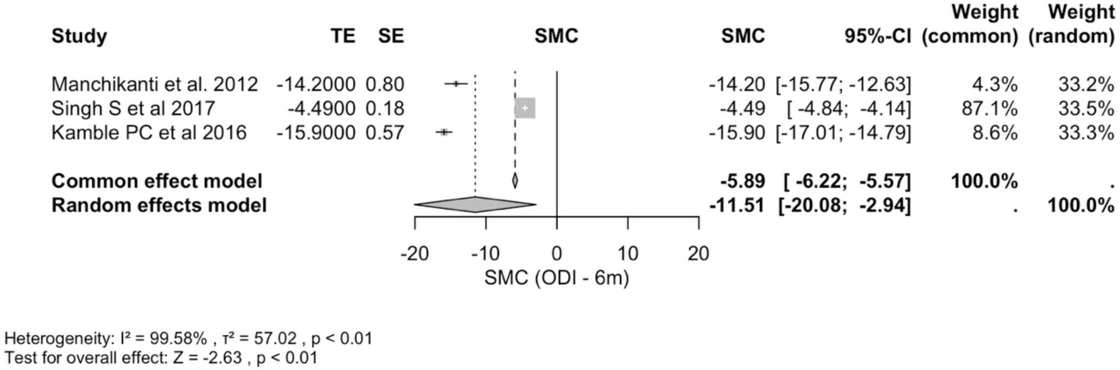

Supplementary Figure 12: ODI Forest Plots for the randomised controlled trials only CESI sensitivity analysis, comparing Baseline to various timeframes

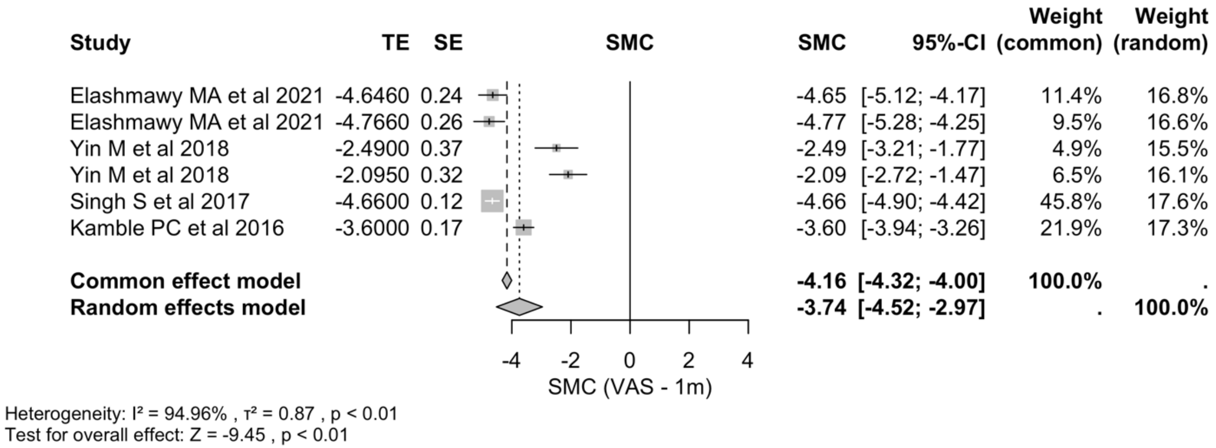

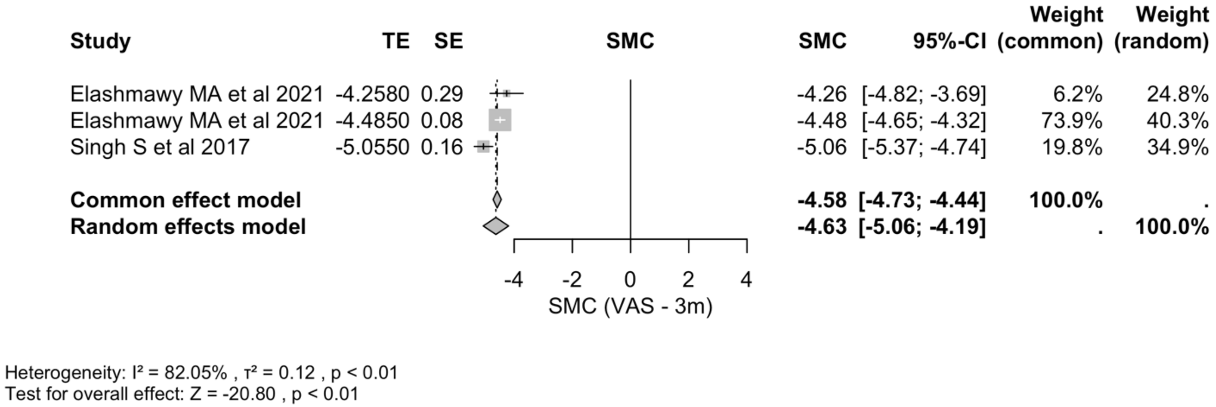

Supplementary Figure 13: VAS Forest Plots for the randomised controlled trials only CESI sensitivity analysis, comparing Baseline to various timeframes

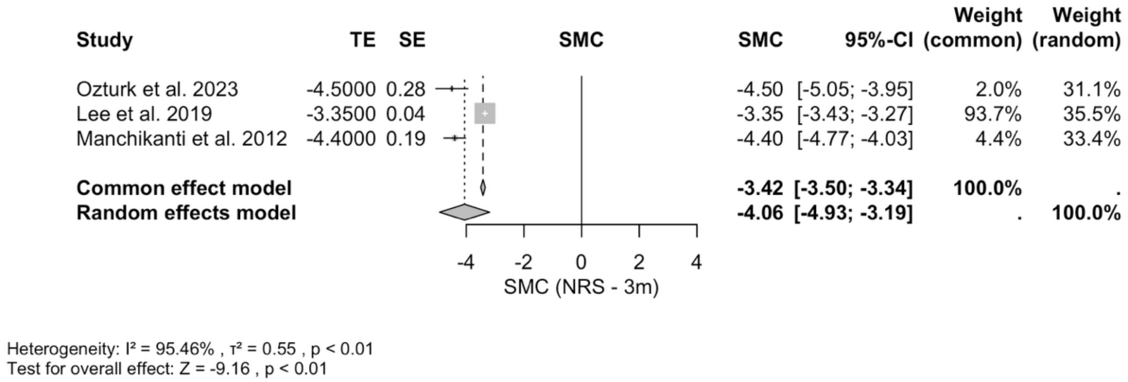

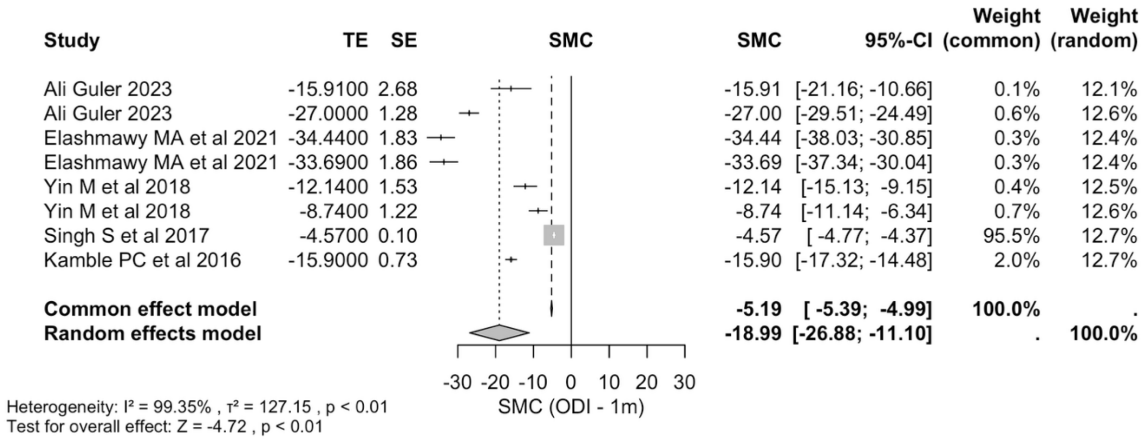

Supplementary Figure 14: NRS and ODI Forest Plots for the main CESI analysis, comparing Baseline to various timeframes

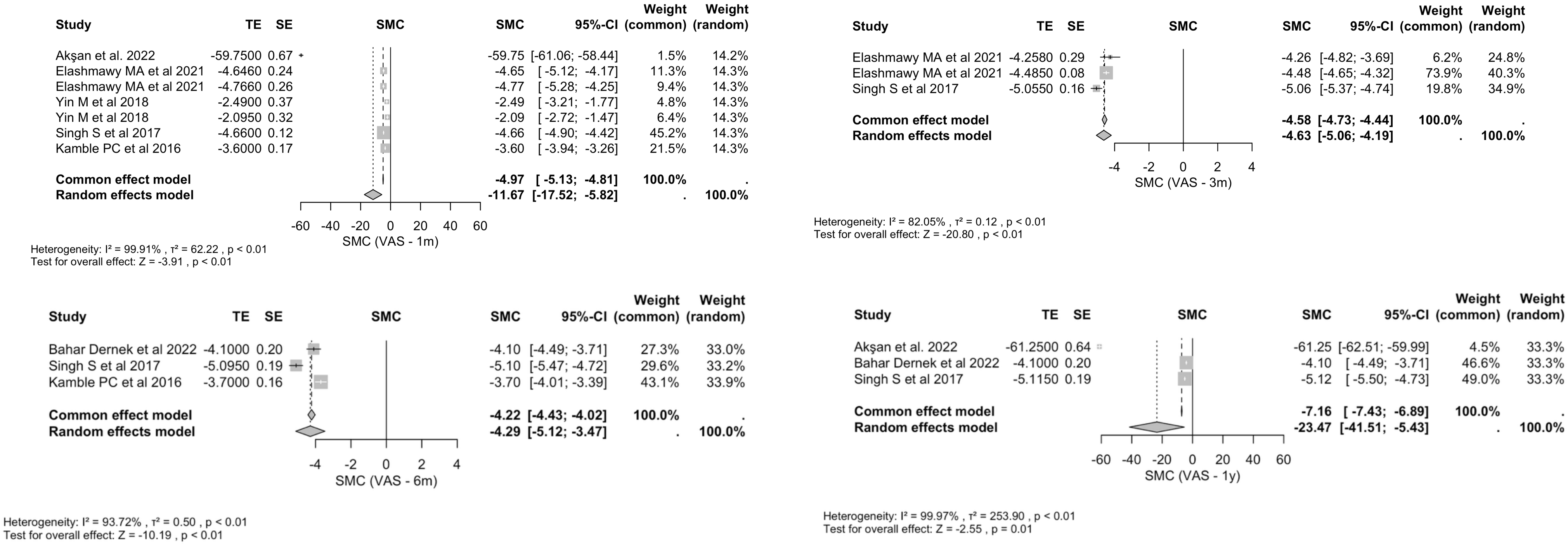

Supplementary Figure 15: VAS Forest Plots for the main CESI analysis, comparing Baseline to various timeframes

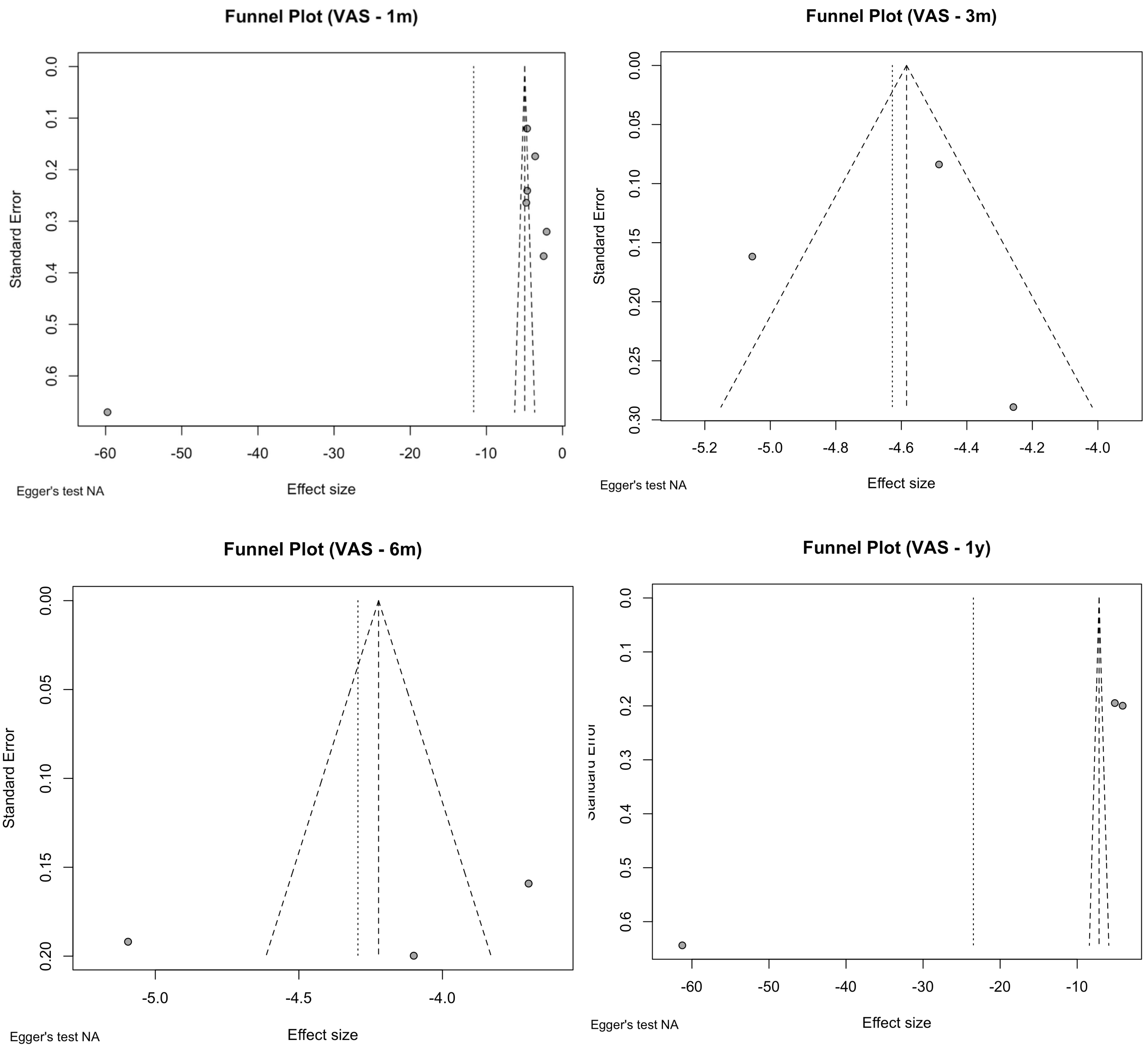

Supplementary Figure 16: Funnel Plot for CESI - VAS

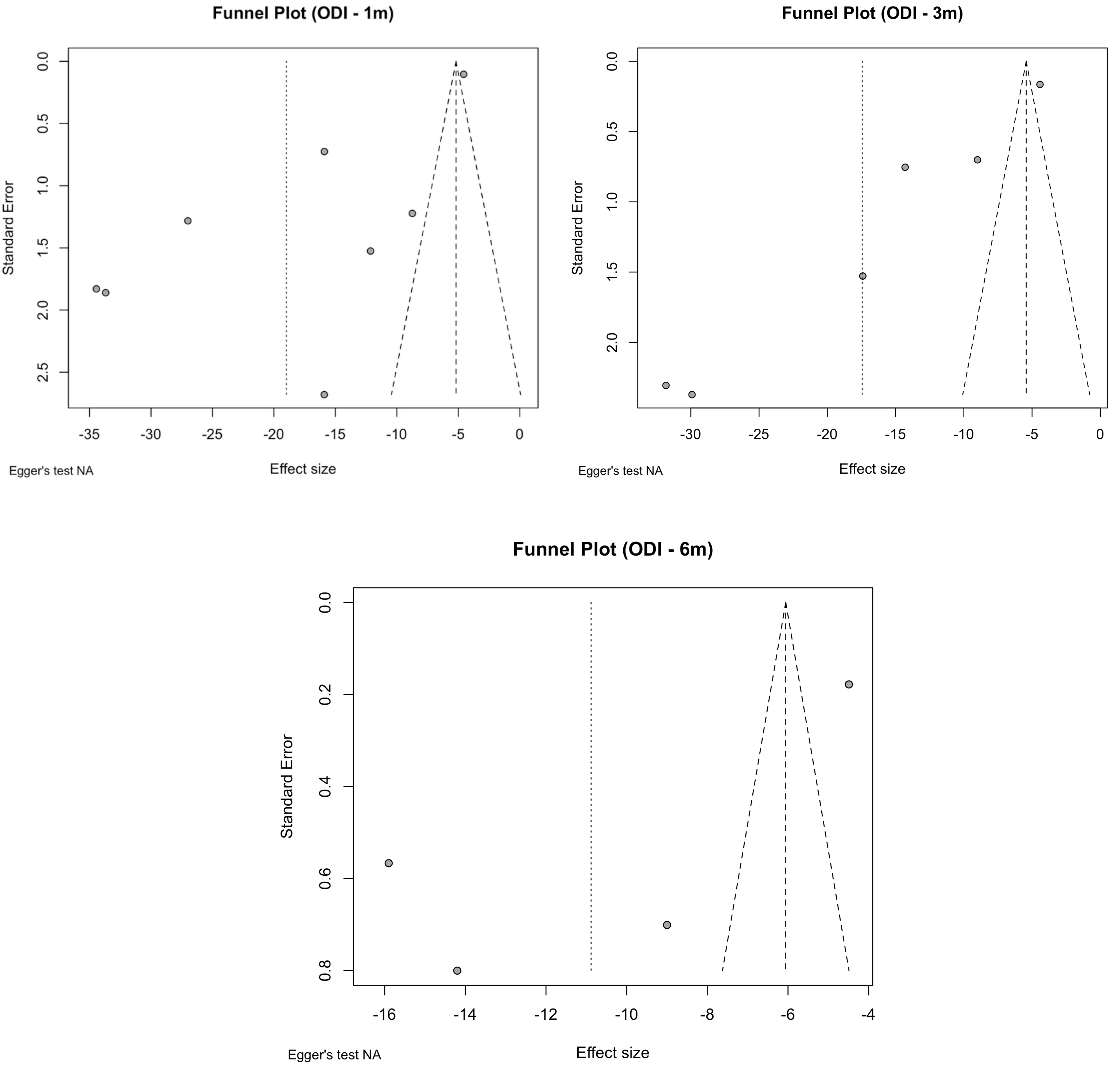

Supplementary Figure 17: Funnel Plot for CESI - ODI

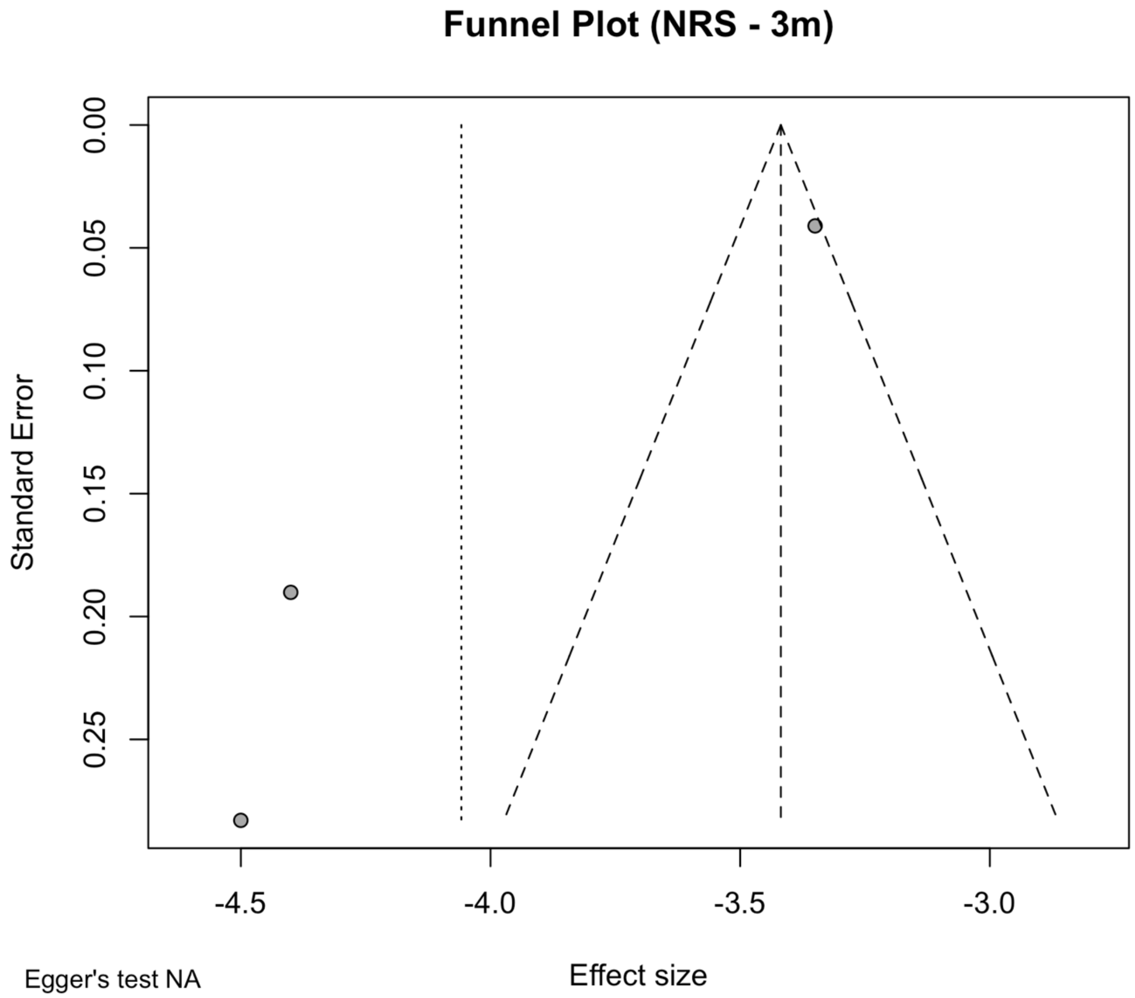

Supplementary Figure 18: Funnel Plot for CESI - NRS

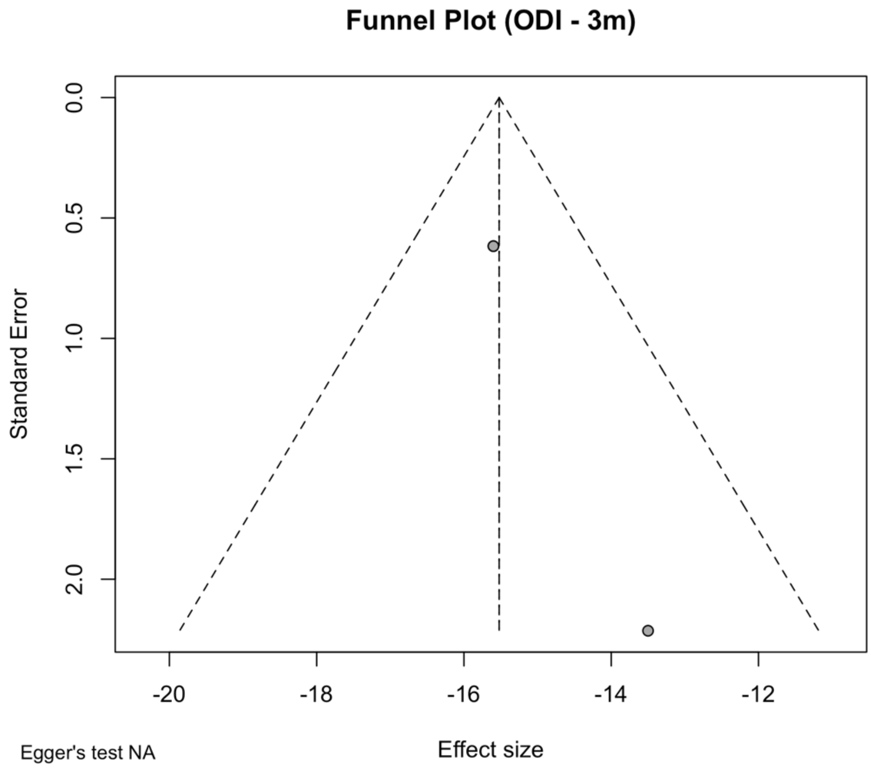

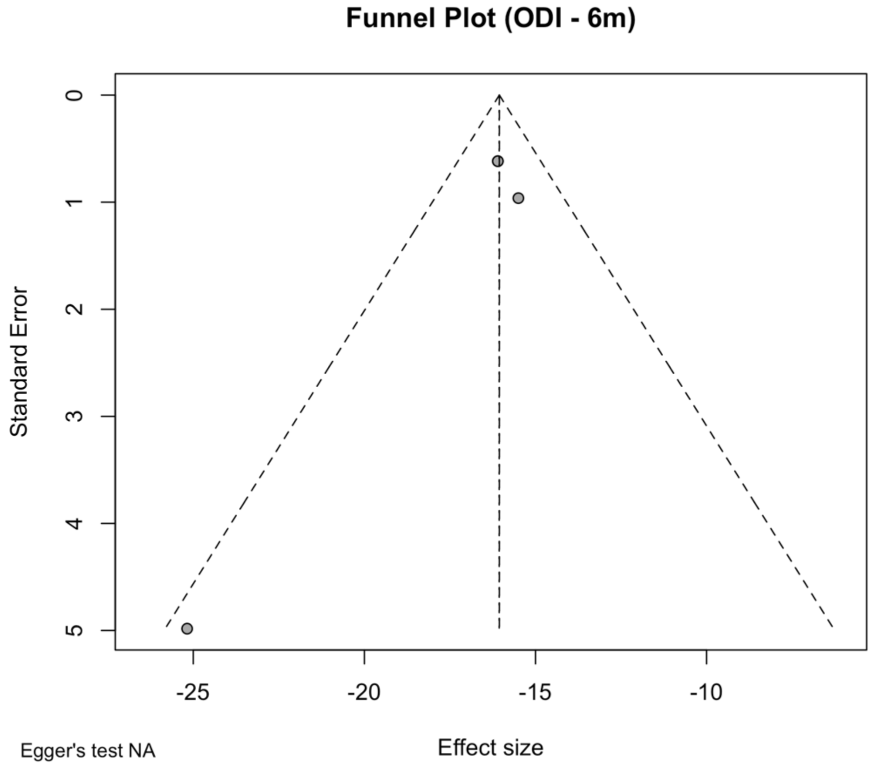

- Supplementary Figure 19: Funnel Plot for CESI - ODI

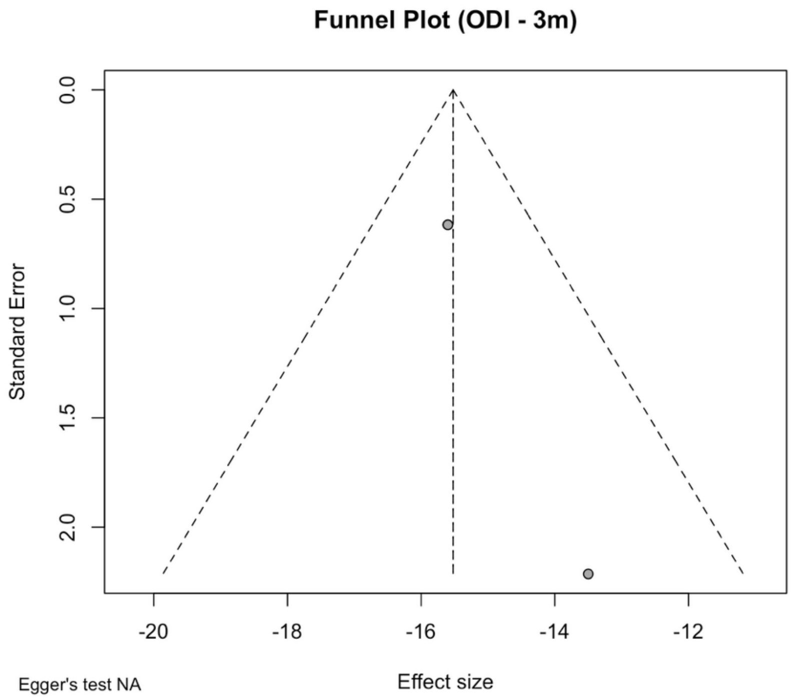

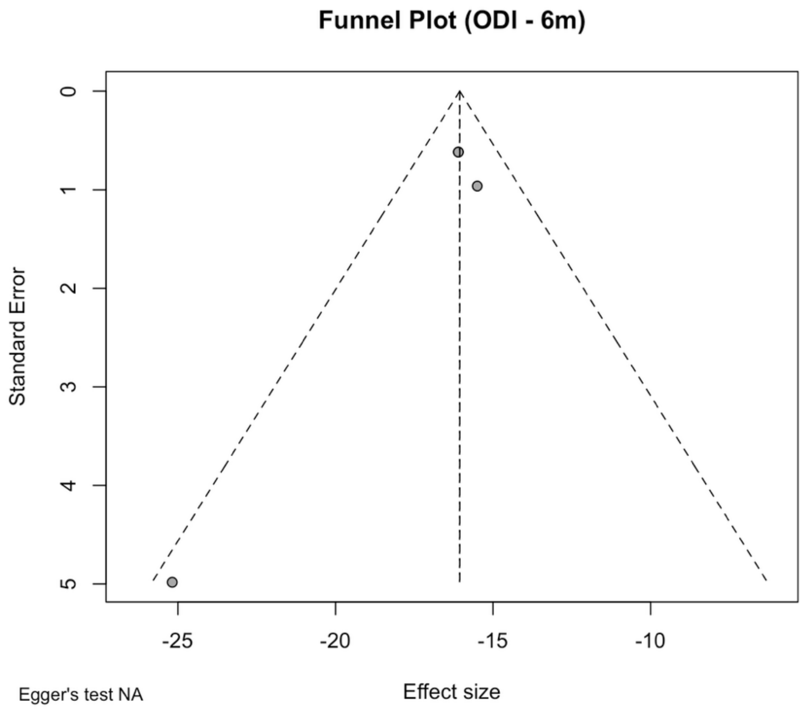

Supplementary Figure 20: Funnel Plot for IESI - ODI

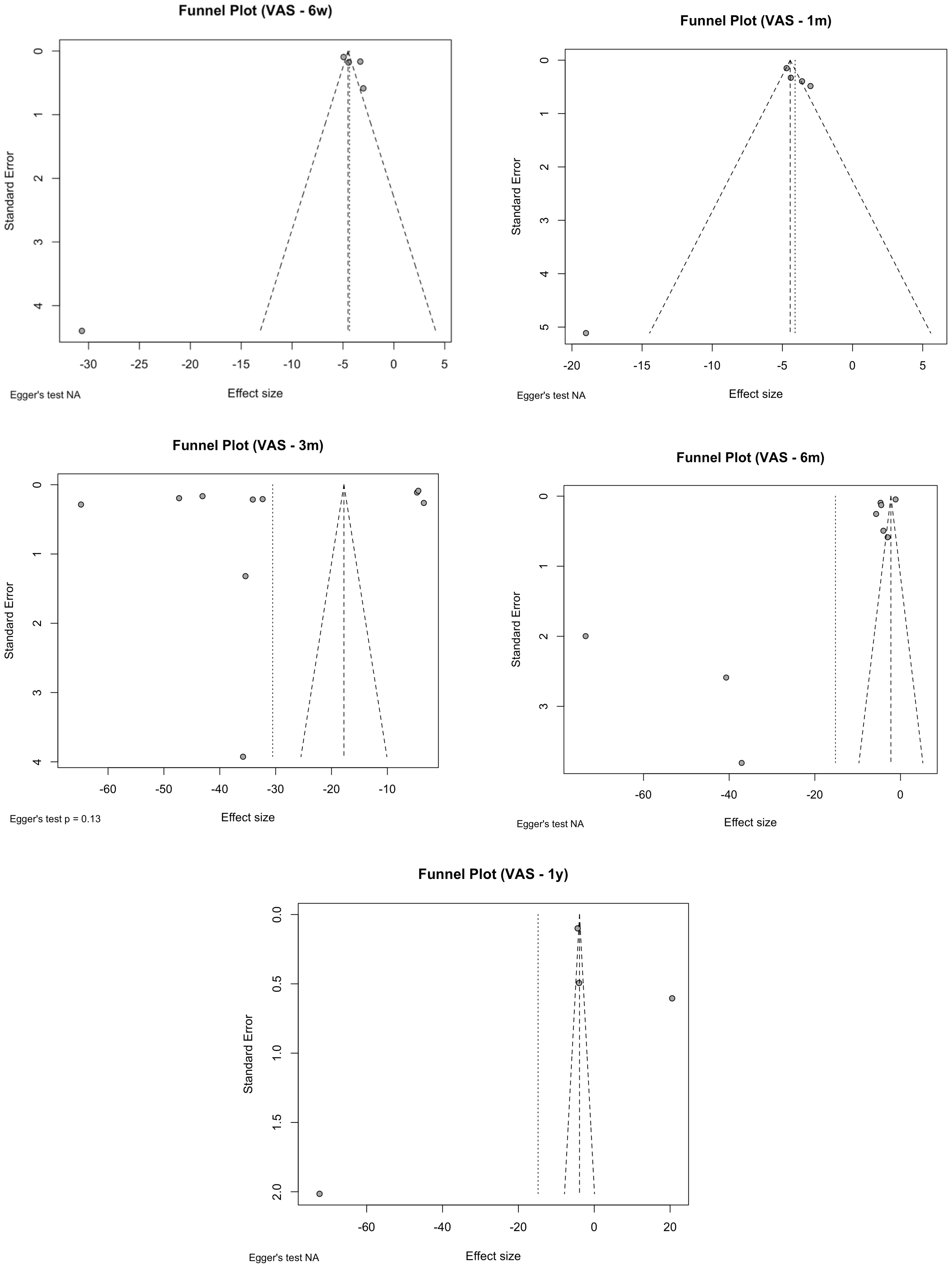

Supplementary Figure 21: Funnel Plot for TFESI - VAS

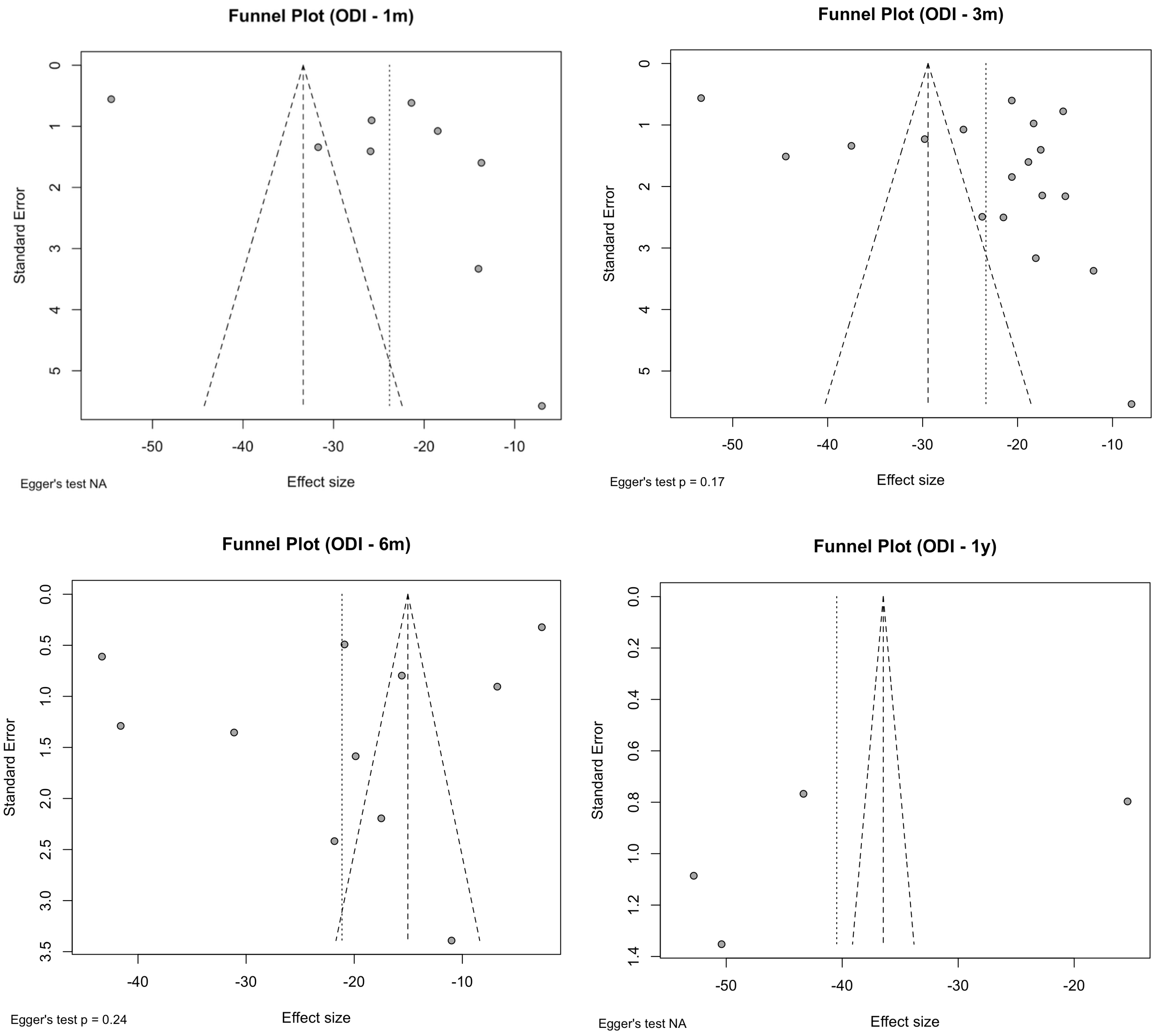

Supplementary Figure 22: Funnel Plot for TFESI - ODI

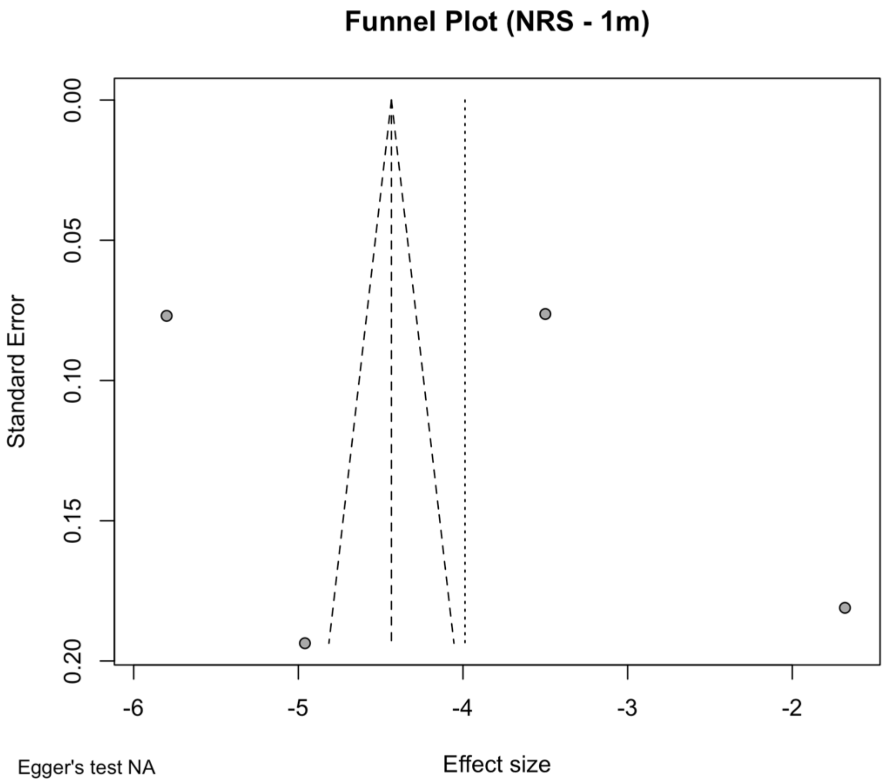

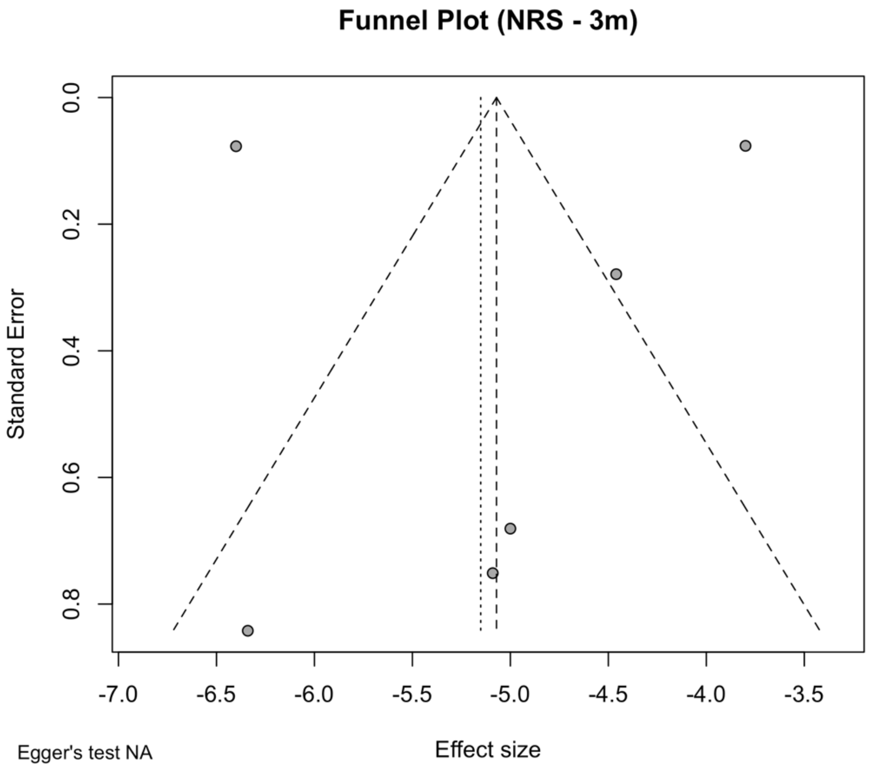

Supplementary Figure 23: Funnel Plot for TFESI - NRS
